# Modelling the regulatory toxicology system to promote the effective use of new approach methodologies: a system thinking approach

**DOI:** 10.1016/j.namjnl.2026.100120

**Published:** 2026-07-24

**Authors:** Angela Bearth, Birgit Kopainsky, Lowenna B. Jones, Gunn E. Vist, Trine Husøy, Camilla Svendsen, Paul Whaley, Sebastian Hoffmann, Heather M.R. Ames, Denise Bloch, Aleksandra Čavoški, Holly G. Davies, Arianna Giusti, Thomas Hartung, Seok Kwon, Olivia J. Osborne, Christophe Rousselle, Jennifer B. Sass, Ulla Simanainen, Kristina A. Thayer, Daniele Wikoff, Gro H. Mathisen

**Affiliations:** a Norwegian Scientific Committee for Food and Environment, Norwegian Institute of Public Health, Oslo, Norway; b HF Partners, Oleanderstrasse 14, 8050 Zurich, Switzerland; c System Dynamics Group, University of Bergen, Bergen, Norway; d School of Sociological Studies, Politics and International Relations, University of Sheffield, Sheffield, UK; e Division for Health Services, Norwegian Institute of Public Health, Oslo, Norway; f Department of Food Safety, Norwegian Institute of Public Health, Oslo, Norway; g Department of Chemical Toxicology, Norwegian Institute of Public Health, Oslo, Norway; h Lancaster Environment Centre, Lancaster University, Lancaster, UK; i Evidence-Based Toxicology Collaboration (EBTC), Johns Hopkins University, Bloomberg School of Public Health, Baltimore, MD, USA; j seh consulting + services, Paderborn, Germany; k Department of Pesticides Safety, German Federal Institute for Risk Assessment (BfR), Berlin, Germany; l Birmingham Law School, University of Birmingham, UK; m Washington State Department of Health, Tumwater, WA, USA; n Cosmetics Europe Avenue Herrmann-Debroux 40, 1160 Auderghem, Belgium; o Center for Alternatives to Animal Testing (CAAT), Johns Hopkins University, Bloomberg School of Public Health, Baltimore, MD, USA; p CAAT Europe, University of Konstanz, Konstanz, Germany; q Department of Pharmacology, Yong Loo Lin School of Medicine, National University of Singapore, Singapore; r Chemical Risk Assessment Team, Science, Evidence and Research Division, Food Standards Agency, Westminster, London, UK; s ANSES/DAEI-French Agency for Food, Environmental and Occupational Health & Safety, 14 rue Pierre et Marie Curie – 94701 Maisons-Alfort Cedex, France; t Natural Resources Defense Council, Washington DC, USA; u Chemicals Agency (ECHA), Helsinki 00121, Finland Directorate of Regulatory Cooperation, Biocides and Water, European, Finland; v Office of Environmental Health Hazard Assessment (OEHHA), Sacramento, CA, USA; w ToxStrategies, Asheville, NC, USA

**Keywords:** Next generation risk assessment, New approach methodologies, Chemical risk assessment, Systems thinking

## Abstract

The term “New Approach Methodologies” (NAMs) comprises a plethora of approaches with the potential for testing the safety, efficacy, and biological effects of chemicals, pharmaceuticals, and other substances without relying exclusively on traditional animal testing methodologies. While it is debateable whether “new” is the appropriate terminology – some NAMs have been around for several decades, while others have been developed recently – many scholars have observed that the effective uptake of NAMs in regulatory toxicology remains a challenge. The interdisciplinary CHANGE project applies a systems thinking approach to this challenge by modelling the behaviour of the regulatory toxicology system shaped by system-level factors and their interconnections. This article reports findings from two three-day workshops held in Oslo in summer 2024 (Workshop 1 “Explore”) and 2025 (Workshop 2 “Reflect”) with experienced practitioners from regulatory agencies, industry, civil society organisations and academia. Through structured collaborative activities, including small- and large-group discussions, modelling exercises and reflection activities, considerable qualitative data was gathered. Most sessions were recorded, transcribed, and analysed using a multi-step thematic analysis explicitly guided by systems thinking principles, enabling the identification of systemic structures and feedback mechanisms and their visualization in the form of a systems model. Insights from Workshop 1 “Explore” were revisited and stress-tested in Workshop 2 “Reflect”, deepening the robustness of the resulting systems model. This systems model shows structural, economic, social, cultural and technical factors that are interlinked through reinforcing and balancing feedback loops that promote or inhibit the effective use of NAMs. The systems model shows potential leverage points for system change, for instance enhancing the stability and reliability of the supply chain or ensuring that actors in the regulatory toxicology system have both educational and institutional support to effectively change established practices.

## 1 Introduction

The global regulatory toxicology system serves to protect human and environmental health through evaluation, communication, and management of potential adverse effects of chemical substances on human health and the environment. Regulatory toxicology can be conceptualised as a complex evolving machine, shaped by the incremental addition of levers, gears and mechanisms over time in response to scientific developments and emerging regulatory challenges. As the awareness of the potential adverse effects on human health and on the environment associated with chemical exposure grew, the infrastructure, processes and tools of regulatory toxicology have also evolved and expanded. Regulatory toxicology generally relies on hazard identification, dose-response assessment, exposure assessment, and risk characterisation to determine acceptable levels of chemical exposure (World Health Organization, 2009). These assessments are often guided by principles and informed by test guidelines developed by recognised organisations (e.g., Organisation for Economic Co-operation and Development) to harmonise testing approaches and data acceptance across jurisdictions. Regional, national and international regulatory agencies (e.g., European Chemicals Agency, U.S. Food and Drug Administration) utilise these guidelines and principles, as well as information from the published literature, to inform approvals, restrictions, bans of chemical substances, risk management, and risk communication.

In general, the machine’s levers, gears and mechanisms are well coordinated, yet some mechanics may have become outdated (e.g., limited scientific justification for dog studies for agricultural chemical and pesticide testing (Box and Spielmann, 2005; Panzarea et al., 2022)) or are not compatible with newer parts (e.g., integration of new methodologies into genotoxicity testing). Updating the machine, in plain words, would entail shifting the risk assessment paradigm from predominantly animal-based testing increasingly towards non-animal-based testing. This might enhance human and environmental protection through higher-throughput testing, cost-effectiveness and a better mechanistic understanding of effects on the human system (Hartung and Tsatsakis, 2021; National Research Council, 2007; Schmeisser et al., 2023), not to mention ethical arguments (Hubrecht and Carter, 2019). Yet this paradigm shift challenges the integral structure of the machine across jurisdictions (i.e., the regulatory system). Despite scientific advances and growing political and societal momentum, the effective uptake of so-called NAMs – referring to New Approach Methodologies or Non-Animal Methodologies, depending on personal and institutional usage – remains a challenge within regulatory toxicology. Evolving the entire system to incorporate NAMs will require coordinated change management, meaning the foresighted process of planning, implementing and reinforcing change that considers challenges, risks and unforeseen consequences (Berggren and Worth, 2026).

In our article, we take a broad stance on NAMs (Schmeisser et al., 2023), defining them as a wide range of technologies and approaches – from *in silico* models to in vitro assays, organ-on-a-chip systems, refined animal testing, and integrated testing strategies – that are continuously advancing. Although no global registry of NAMs discovered, developed, and in use for regulatory decision making exists, only a few have been formally adopted in regulatory frameworks (e.g., non-animal assays for skin sensitisation or eye irritation, in vitro assays for genotoxicity) (Carramusa et al., 2024; Hartung and Tsatsakis, 2021; OECD, 2021). A multitude of publications have deplored the slow and inefficient uptake of new scientific developments in regulatory toxicology (Carmichael et al., 2022), others have called for a careful transition or cautioned against an ineffective use of NAMs (Jaeschke and Ramachandran, 2025; National Academies of Sciences, 2022). Generally, NAMs are currently primarily applied for early-stage screening, hazard identification, and priority setting, but not effectively for decision making regarding safety in a regulatory context. Inconclusive NAM-based results might still be followed up by in vivo testing. This observation highlights that NAMs, possibly, are not currently used to their full potential in risk assessment. Effective use of NAMs means that the regulatory toxicology system reliably discovers, releases, recognises, endorses and uses fit-for-purpose, scientifically robust NAMs that provide actionable evidence for decision making, while excluding NAMs that do not (yet), thereby improving efficiency, maintaining and improving protection levels, and reducing animal testing.

This is not the first article to explore the barriers and drivers of the effective use of NAMs in regulatory toxicology; manifold articles have been published that focus on the technical, economic, legal, or psychological challenges and opportunities (Bearth, Roth et al., 2025; Čavoški et al., 2025; Mondou et al., 2020; Pain et al., 2020; Sass et al., 2024). A recent article (Berggren and Worth, 2026), based on informal conversations with a heterogeneous group of stakeholders, identified single change management strategies, such as building confidence and trust and evolving collaboration models among important stakeholders. A recently published commentary (Jeffcoat et al., 2025) introduced systems thinking to the field, in the form of the “toxic ignorance cycle.” This self-reinforcing cycle implies that knowledge gaps in toxicological data and emerging tools and technologies foster institutionalised ignorance, leading to non-decision making and regulatory inaction that ultimately, reproduces the knowledge gaps.

The present article builds on these observations of the regulatory toxicology system by embracing a more holistic perspective, co-creating a complex adaptive model of regulatory toxicology within which system interventions and their consequences can be modelled with experienced system actors. This article builds on a prior publication (Bearth, Kopainsky et al., 2025) that utilised the socio-technical systems (STS) model (Davis et al., 2014; Hendrick, 2006) to structure the regulatory toxicology system into infrastructures, processes, culture, technology, goals and actors. The article’s core findings were that currently, neither the technical aspects (infrastructure, processes, or technology), nor the social aspects (cultures, actors or goals) fully support the effective use of NAMs (Bearth, Kopainsky et al., 2025). To build our complex adaptive model of regulatory toxicology, systems thinking is applied. Systems thinking is a holistic approach to describing the interconnections between the components of a system (Meadows, 2008). System behaviours, meaning the mechanisms at work within the regulatory toxicology system, arise from interactions between different barriers and drivers, across various actors with varying goals and objectives, and beyond the limits of individual siloes. System behaviours describe patterns of outcomes that a system produces over time, emerging from its elements and their interconnections. For our purposes, a systems model was developed that includes a stocks-and-flows model and several feedback loops. The progression of NAMs from discovery to use is visualised in the stocks-and-flows model, while the feedback loops identify system components and highlight how these influence the systems’ behaviour. Utilising this mode of thinking, the goal of this article is to model the current state of the regulatory toxicology system (i.e., the machine’s parts and how they interact with each other) and identify leverage points for system change in context of the effective use of NAMs in decision making. The systems model aims to highlight where the machine’s mechanisms are not working according to their intended functionality, where levers and gears could be replaced, enhanced or updated, and which new parts are needed to enhance the effective use of NAMs.

## 2 Methodological approach

### 2.1 The CHANGE project

This study was conducted as part of the Collaboration to Harmonise the Assessment of Next Generation Evidence (CHANGE) project (Mathisen et al., 2024). The CHANGE project is rooted in systems thinking and is concerned that NAMs, despite being developed at a relatively quick pace, currently are not used to their full potential in regulatory toxicology. The project’s goal is to design system-level interventions that promote the effective use of NAMs in the regulatory toxicology system (Bearth, Kopainsky et al., 2025; Mathisen et al., 2024). Intervening on a socio-technical system requires understanding how its parts interact, meaning how system-level factors contribute to the effectiveness of use of NAMs. To do this, the CHANGE project has adopted an interdisciplinary co-creation methodology that involves several in-person and online workshops with international experts in the field, led by an interdisciplinary project group, and guided by an international advisory board whose members have extensive experience in regulatory toxicology.

### 2.2 Study design

**Fig. 1:**
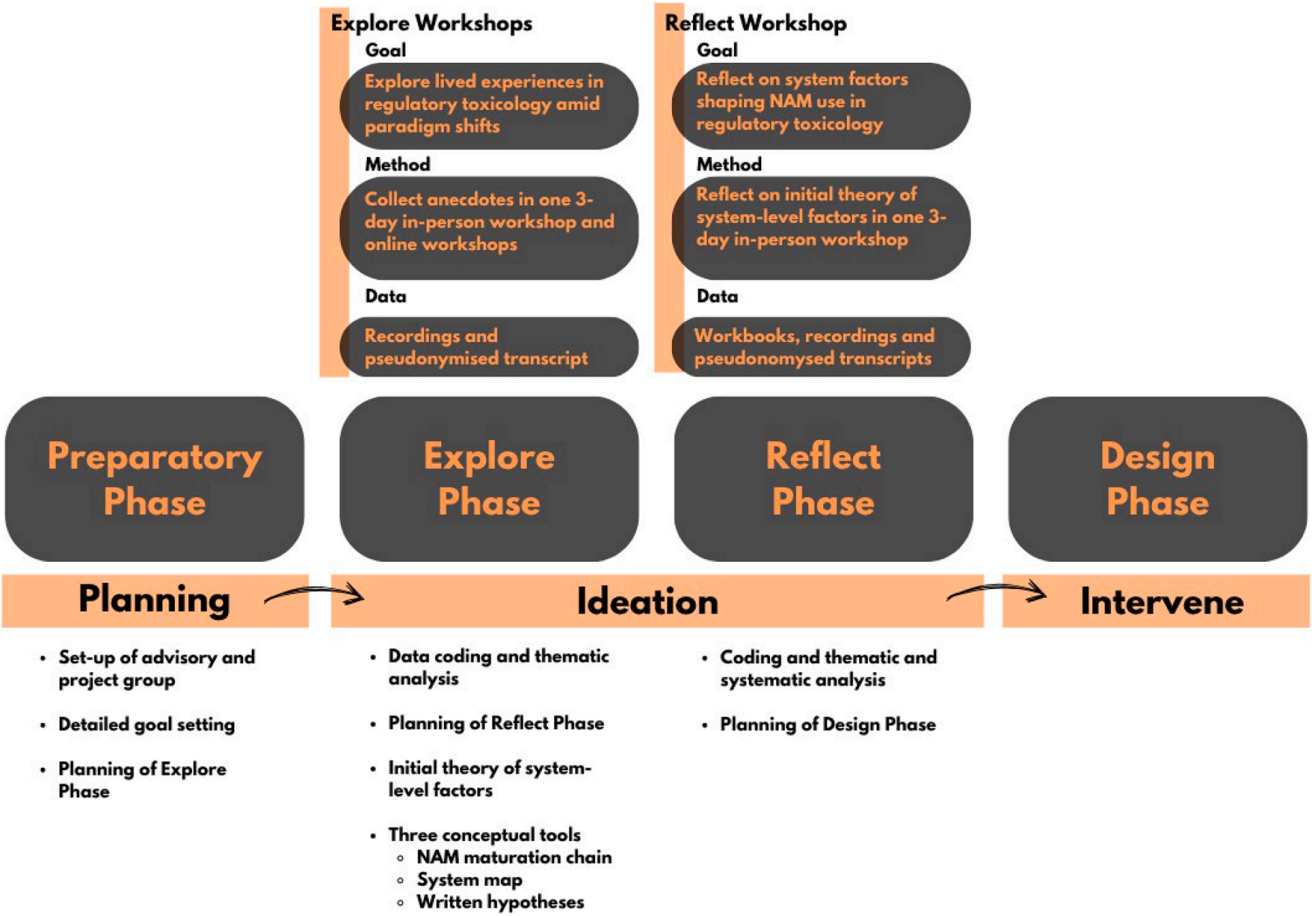
Overview of study design.

The data for the model presented in this article was collected in two three-day in-person workshops, held in Oslo (Norway) in summer 2024 and 2025, and in a series of online workshops in autumn 2024. The workshops featured a heterogeneous group of international participants with in-depth and diverse experiences of working in the regulatory toxicology system (i.e., risk managers, risk assessors and researchers from academic, industry, non-governmental organisations, regulatory institutions, from varying disciplines and legal jurisdictions). Workshop participants were recruited through various complimentary strategies: 1) asking the international advisory board for recommendations (with members from the United States of America, Asia, Australia, Europe), 2) identifying and inviting stakeholder groups of interest by institution (i.e., WHO, OECD, ILMERAC), sector (i.e., regulatory authorities, scientific organisations, academia, industry and SMEs) and geographical region (i.e., participants from all continents), 3) via snowball technique (i.e., workshop participants recommendations or direct invitation). The majority of participants took part in both workshops, while some only attended one or the other. To facilitate further participation from industry, as well as Asia, South America, and Oceania, online workshops were organised (cf. Table B1 in Appendix B for an overview of sectors and geographical regions of the participants). The first workshop (“explore”) focused on collecting data about the structure of the regulatory toxicology system and participant’s lived experiences amid paradigm shifts. The second workshop (“reflect”) was focused on developing theories about the system behaviour from the data of the first workshop. Fig. 1 provides an overview of the study design, specifically the phases and workshops that contributed to the data collection for our model.

**The first in-person and online workshops (i.e., “explore” phase)** explored lived experiences of people working in the regulatory toxicology system through anecdotal storytelling by the workshop participants. These anecdotes were collected in group discussion sessions, that were recorded, pseudonymised, transcribed and coded into a flexible code-scheme. Thematic analysis was applied to this data to form an initial theory of system-level factors that inhibit or promote the effective use of NAMs in regulatory toxicology. After the “explore” workshops, the initial theory of system-level factors that inhibit or promote the effective use of NAMs in regulatory toxicology was further developed through discussion and other collaborative exercises in weekly meetings with the project team. This initial theory was then translated into three conceptual tools for the second in-person workshop. In the “reflect” phase those would evolve into the system model presented in this article: an initial draft of the *New Approach Methodology maturation chain* (“NAM maturation chain”) that visualises a NAMs path from discovery to adoption for regulatory decision making; an initial draft of the *systems model;* and a series of *written hypotheses* (see Supplemental Material). Detailed information on methodology and results of the first workshop and follow-up workshops is published elsewhere, specifically coding process, analytical framework and validation procedures, (Bearth, Kopainsky et al., 2025; Diemar et al., 2025; Jones et al., 2025; Mathisen and Solstad, 2025).

**Fig. 2:**
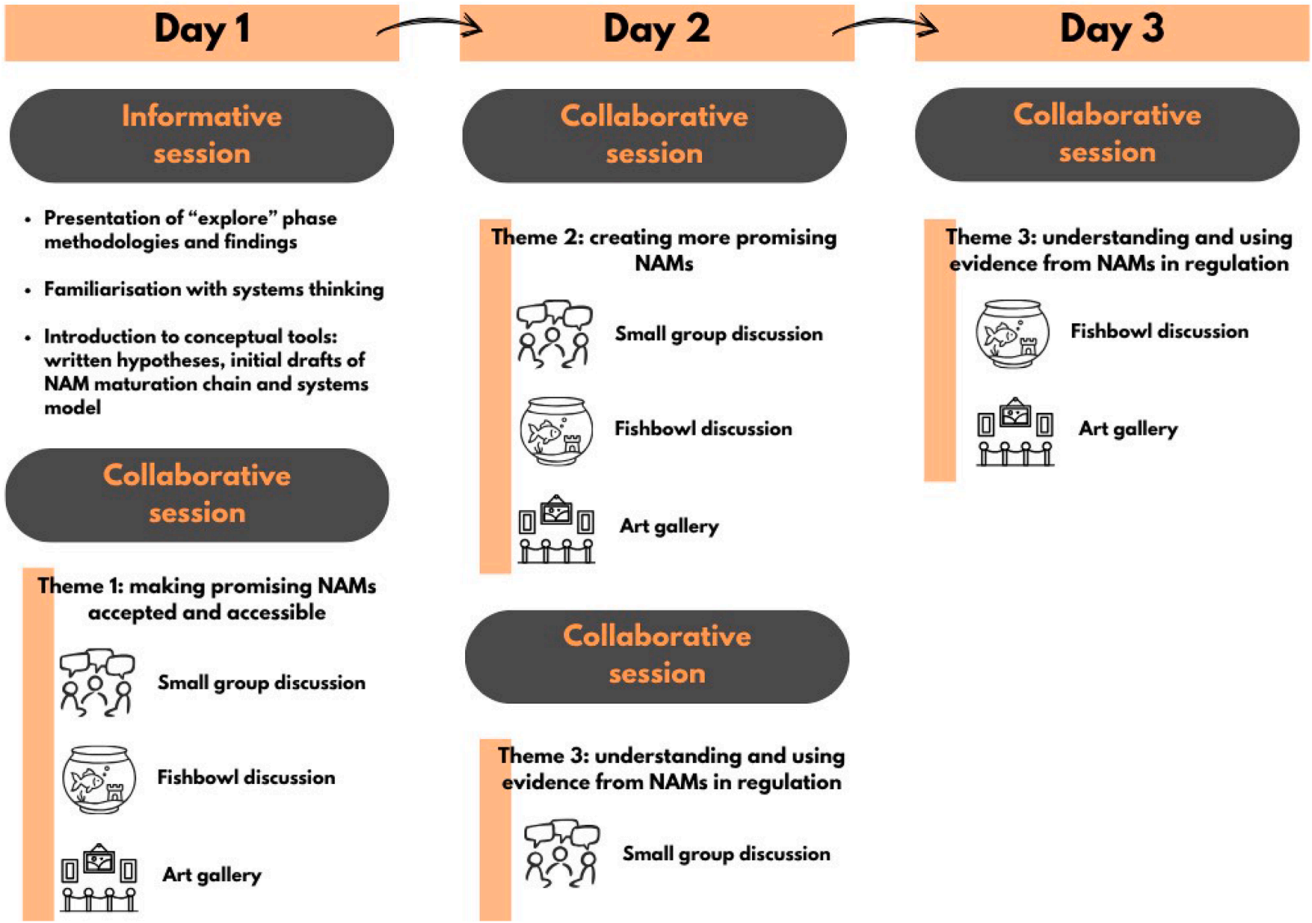
Overview of second workshop (i.e., “reflect” phase).

**The second workshop (i.e., “reflect” phase)** reflected on the initial findings of system-level factors that inhibit or promote the effective use of NAMs in regulatory toxicology. It comprised informative and collaborative sessions; Fig. 2 offers an overview of the basic workshop structure. In the informative session at the start of the workshop, the methodologies and findings from the “explore” phase were presented and the participants were familiarised with systems thinking and the approaches. Further, they were introduced to the conceptual tools used in the workshop, specifically the written hypotheses, the initial drafts of the NAM maturation chain and the systems model. The subsequent collaborative sessions were organised under three themes, focusing on different parts of the initial draft of the NAM maturation chain: Theme 1: making promising NAMs accepted and accessible; Theme 2: creating more promising NAMs; Theme 3: understanding and using evidence from promising NAMs in regulation.

Each theme consisted of between three and four overarching hypotheses with two to six sub-hypotheses about the system factors promoting or inhibiting the effective use of NAMs. To support discussion, participants received printouts of the written hypotheses serving as main discussion tool. The discussions were further supported by printouts of the initial drafts of the NAM maturation chain and systems model. Once per theme, three times throughout the workshop, the collaborative sessions were run, following this sequence: i) small group discussions (60 min, seven groups in parallel, 6–7 randomly assigned participants per group); ii) fishbowl discussions (50 min, two groups in parallel, 25–30 randomly assigned participants per group), and iii) art galleries (35 min, individual work). Randomisation was done through an online randomisation tool (i.e., https://www.randomlists.com/team-generator). Specific measures were taken to consider cognitive and social biases and dynamics before and during these sessions (e.g., dominant voices, consensus effects), specifically a) piloting of methodology and training of moderators prior to the workshop, b) encouragement of a collaborative atmosphere among participants through initial input session, c) active invitation of less dominant participants to provide their inputs, d) inclusion of individual work in addition to collaborative sessions (i.e., art gallery session), and e) remixing of groups between discussion sessions to prevent undesirable social dynamics becoming embedded. These approaches are discussed in more detail in two prior publications, one from the perspectives of the project group and one from the workshop participants (Diemar et al., 2025; Jones et al., 2025). For the small-group discussions, to make sure that all hypotheses for a given theme were covered in the small group sessions, each group received a set of randomly assigned hypotheses. The moderators read these hypotheses aloud and passed them out in written form. Participants could propose changes either verbally or directly on paper by sketching on a table-side NAM maturation chain. Afterwards, these sketches were displayed on the walls of the main workshop room.

The fishbowl discussions took place directly after the small group discussions. The fishbowl discussions featured a moderator, an active discussion panel and a passive audience with participants being able to move freely from panel to audience and vice versa. The fishbowl discussion approach is presented in more detail elsewhere (Jones et al., 2025). All fishbowl discussions were recorded using iPhone-recordings and transcribed using Whisper (OpenAI, 2024).

In the art gallery session, each participant received a theme-specific workbook (Andersen et al., 2012) and was invited to fill out the workbook and add notes to the sketches from the small group discussions. The workbook can be found in the Supplemental Materials. The workbook comprised an overview page with all hypotheses and sub-hypotheses belonging to each theme, one closed-format question about their agreement with the individual hypothesis (response options: strongly disagree, disagree, agree, strongly agree and do not know) and one comment field per hypothesis/sub-hypothesis. As for the first workshop (i.e., “explore” phase), the formal program was supported by a rich social program designed to create a pleasant atmosphere for building trust and social relationships that would encourage open sharing (Jones et al., 2025).

### 2.3 Data preparation and analysis

Data preparation and analysis took place after the second workshop. The data for this analysis were 144 digitised workbooks (3 themes * 48 participants, typed up by GM), six fishbowl discussion transcripts, and 21 annotated sketches from the small group discussions. The data preparation and analysis were led by two researchers: AB with a background in social science and risk research and BK with a background in systems thinking. AB and BK worked separately through the digitised workbooks, utilising them to iteratively refine the systems model (BK) and the series of hypotheses (AB). Specific tools served to ensure that the system model and hypotheses were firmly rooted in the data, most importantly a table that recorded the original quote(s) associated with a variable in the system model or change in the hypotheses, following the procedure outlined in Eker & Zimmermann (2016). Feedback loops were continuously detected by checking whether chains of variables formed a closed circuit. Regular exchanges were scheduled to ensure consistency in data preparation and analysis. The other members of the project group (GM, LJ, SH, PW, HA, GV, TH, CS) were invited to continuously comment on the refined systems model and hypotheses, which ensured that the individual perspectives of the two main analysts, AB and BK, were regularly challenged. Two researchers (GM, LJ) coded the six fishbowl discussion transcripts in parallel, utilising the hypothesis structure as a code scheme. One researcher (AB) resolved conflicts between the two separate coded solutions, and BK and AB used the coded transcripts to complement the refined systems model and hypotheses. Lastly, AB went through the small group discussion sketches to ensure nothing was missed.3. Results

Our analysis produced a model of NAM maturation from discovery to use in regulatory decision making (i.e., the “NAM maturation chain”) and a model of interacting factors that govern the effectiveness of use of NAMs in regulatory toxicology (i.e., the “system model”). The system model identifies 16 feedback loops that show how the system factors interact with the maturation chain to cause or prevent the effective use of NAMs. Finally, the 16 loops fit under seven mechanisms that can be used to structure the design of system-level interventions for improving the effective use of NAMs in regulatory toxicology: i) disconnected systems, ii) the NAM innovation-regulation bottleneck, and ii) the early mover hesitancy reinforcement.

### 2.4 The NAM maturation chain

**Fig. 3:**
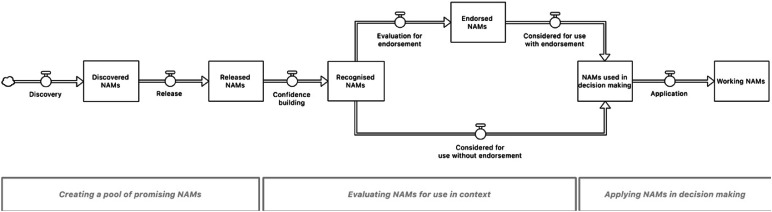
The NAM maturation chain (boxes represent stocks; arrows with valves represent flows). A stock is the accumulated difference between inflows and outflows at a given time point (e.g., the “Discovered NAMs” stock represents all NAMs that are discovered but have not been made available outside of the entity that discovered them). A flow represents the rate at which a stock changes over time, reflecting the inflow and outflow from a stock (e.g., the “Release” flow represents the rate at which discovered NAMs are made available to other entities).

The NAM maturation chain is a model for the progression of NAMs from initial discovery through evaluation to use in decision making (Fig. 3).

In the NAM maturation chain, the abbreviation “NAMs” means either an individual NAM, or multiple individual NAMs combined in a defined manner (e.g., testing battery, assay, modelling approach). The NAM maturation chain is the core structure of the systems model, and it retained the three stages from its initial draft version from the “explore” phase: creating a pool of promising NAMs; evaluating NAMs for use in a context; and applying NAMs in decision making. The NAM maturation chain takes the form of a stock-and-flow model and visualises how the rate of the flows regulates the maturation of NAMs, with each flow acting as a tap that controls release of NAMs from a previous stock into the next stock (Sterman, 2002). The model concerns effective use of NAMs because it is interested in the extent to which:

• NAMs discovered and released are promising: the extent to which NAMs are discovered and released that will ultimately lead to a more effective regulatory toxicology system);
• Promising NAMs become accepted through evaluation: the extent to which NAMs are validated and are shown to be useful for regulatory decision making for a given context
• Validated NAMs are applied to support better decision making: the extent to which NAMs support timely, protective decisions by actors using NAM data (i.e., data for regulatory use or decision making, generated using NAMs)

The system behaviour increases or decreases the flow of NAMs from one stock to the other, allowing or preventing NAMs from moving down the maturation chain toward use in decision making. Note that attrition and recirculation flows (NAMs that leave the NAM maturation chain or flow backwards) are not shown to prevent overcomplicating the visual.

The first section of the NAM maturation chain, “creating a pool of promising NAMs” concerns the tendency of NAM discovery and release toward more rather than less effective use of NAMs. The creation processes start with the “Discovery” flow and contains the stocks “Discovered NAMs” and “Released NAMs,” which are connected through the “Release” flow. Discovery is the invention of new NAMs and is driven by research and innovation in academia, industry and research organisations. It is a process that is internal to an organisation or organisational unit. Examples include how a university lab may be developing a NAM for doing academic research, and how a contract research organisation may develop specific testing methods to market to prospective customers. Such NAMs may or may not ever be released beyond their creating organisation or organisational unit. Release is the making available of a NAM outside the immediate organisation or organisational unit that created the NAM. Release mechanisms not only include scientific publications, presentations at scientific conferences, preprints, and databases, but also marketing materials, direct delivery to an institution that funded or commissioned the NAM, and even release of a NAM from a research and development unit of a company to another unit.

The second section of the NAM maturation chain, “evaluating NAMs for use in a context” pertains to building confidence in and evaluating promising NAMs. Confidence building and evaluation could be achieved through any type of testing, process or assessment approach to find out how well a NAM works for a given purpose. Examples include providing consistent data for a specific regulatory need, or as a screening tool for identifying when a substance may have an undesirable property. (e.g., for a specific regulatory need or information requirement). It begins with the “Confidence building” flow, which represents the organic or targeted process of building confidence in NAMs through better understanding their characteristics (e.g., robustness, relevance, reliability, reproducibility, utility, mechanistic understanding, applicability domain). This flows into the stock of “Recognised NAMs” (i.e., NAMs that have achieved a threshold of recognition, credibility, or confidence for a specific context, NAMs that have gained the attention of notable institutions, NAMs that are recognised as potentially promising for regulatory toxicology).

Next, the NAM maturation chain splits off into two separate branches. The first branch describes the process that leads to the endorsement of NAMs for a specific application by a recognised organisation, via the flow of “Evaluation for endorsement” and the stock of “Endorsed NAMs.” Evaluation for endorsement involves the formal validation of a NAM, involving rigorous testing and often experimental work to assess a NAM’s relevance and reliability, peer-review for scientific acceptability by a panel of reviewers, and regulatory endorsement (i.e., official endorsement by a recognised national or international body, such as the OECD, US EPA, FDA). The stock of “Endorsed NAMs” is drained by the flow “Considered for use with endorsement” (not all endorsed NAMs are equally appropriate for all uses in a context). The second branch consists of the flow “Considered for use without endorsement.” NAMs passing through this flow are not going through an official endorsement process; rather, these NAMs are being applied case-by-case as their utility is being determined for a specific decision making context; they are directly considered for use, without waiting for an official process of endorsement.

Both branches flow into the stock of “NAMs used in decision making", creating a combined pool of NAMs that are being used that have official endorsement, along with NAMs that are being used that do not have official endorsement. As these NAMs are repeatedly applied (the “Application” flow), some will be found to work, and some not. Those that are found to work fill the “Working NAMs” stock. Those are the NAMs that are regularly and routinely applied in decision making in specific contexts at various levels. At regulatory level this will include mandated tests and supporting evidence in dossiers, while at a company level this would include screening compounds for potentially toxic properties. This is the third and final section of the NAM maturation chain.

### 2.5 The systems model: mechanisms and feedback loops

The systems model consists of interrelated variables that increase or decrease rates of flow between stocks and are influenced by the levels of NAMs in the stocks. We categorised the interrelated variables under six overarching mechanisms that describe, in terms of 16 feedback loops, how the regulatory toxicology system currently operates in relation to the effective use of NAMs. All variables, their interconnections, and the resulting feedback loops are firmly grounded in empirical data. The selection of the six overarching mechanisms and 16 feedback loops was a result of intensive and iterative discussions in the project group and with the advisory board. In this section, we introduce these six mechanisms, followed by an in-depth description of each of the specific feedback loops within each mechanism in the subsequent section.

**Fig. A1:**
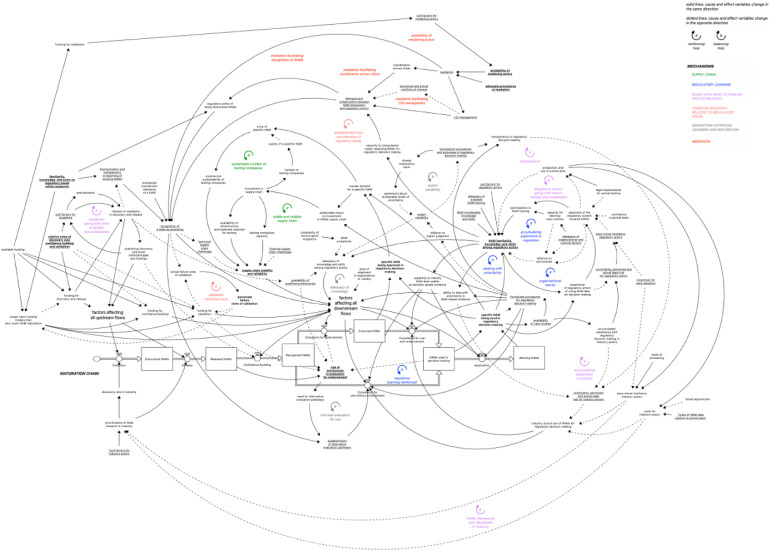
Full system model of regulatory toxicology including all mechanisms and feedback loops.

Feedback loops are circular chains of cause-and-effect relationships in which a change in one variable affects others, which in turn feed back to influence the original variable. Each overarching mechanism consists of several such loops that involve different system actors and operate at different stages of the NAM maturation chain. Fig. A1 in Appendix A presents the systems model in full. Several social and political factors (e.g., political and societal pressure to phase out animal testing or to ensure chemical safety, societal trust in involved institutions) do not directly influence the NAM maturation chain but rather shape the regulatory toxicology system by impacting variables within the overarching mechanisms. A specific example for a social factor impacting the system model is the negative associations and affect people experience towards animal testing (Bearth, Wiesner et al., 2024), which contributed to the submission of the European citizens’ initiative “Save Cruelty Free Cosmetics – Commit to a Europe Without Animal Testing,” and sparked incentives for early adoption of NAMs in the cosmetic sector (European Citizens’ Initiative, 2023). While acknowledging that these social and political factors are relevant, for simplicity purposes, we are not representing them in the subsequent figures or introducing them in this article.

**Fig. 4:**
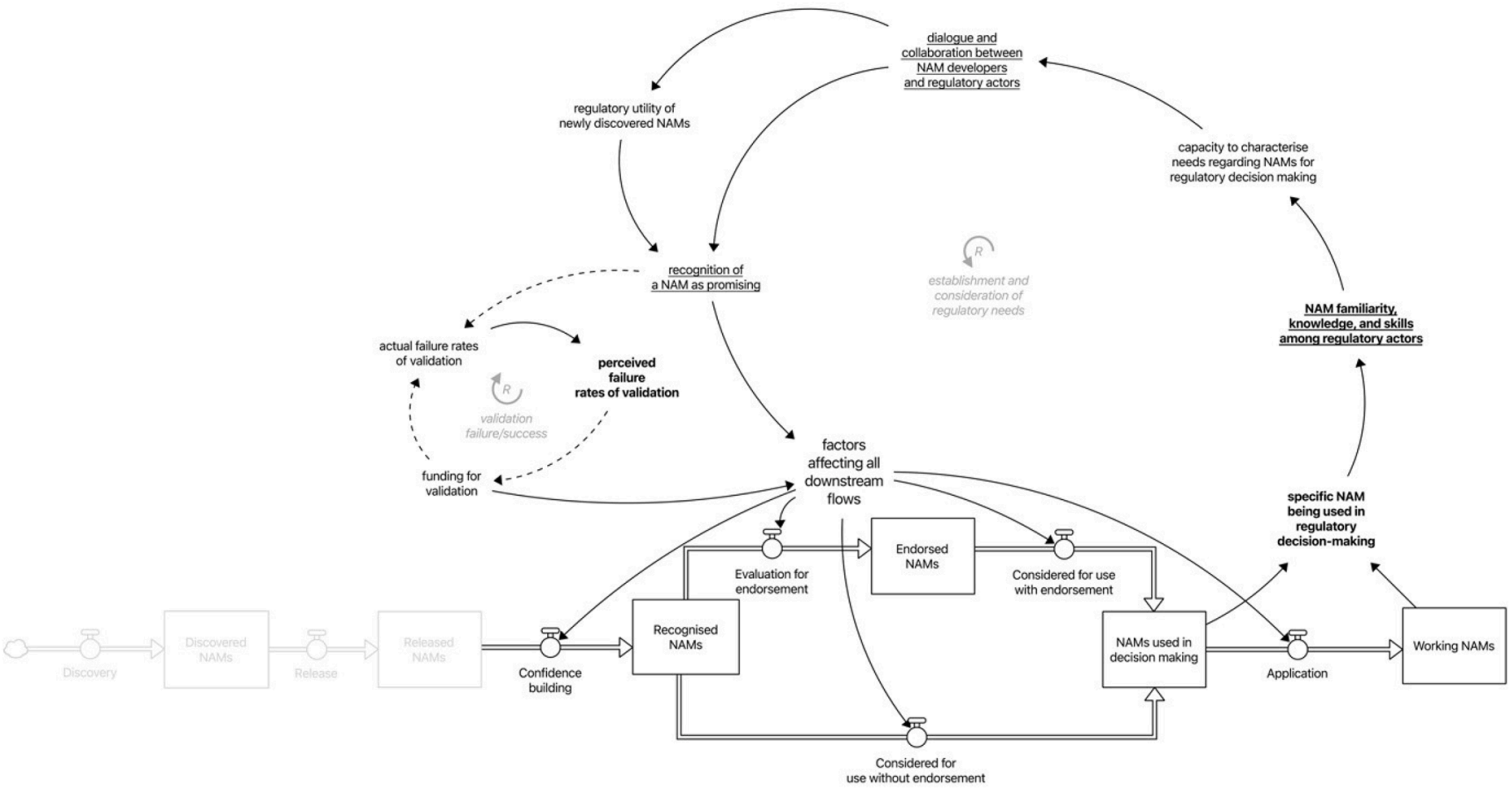
Section of the systems model that shows the “Targeted Research Related to Regulatory Needs” mechanism.

**Fig. 5:**
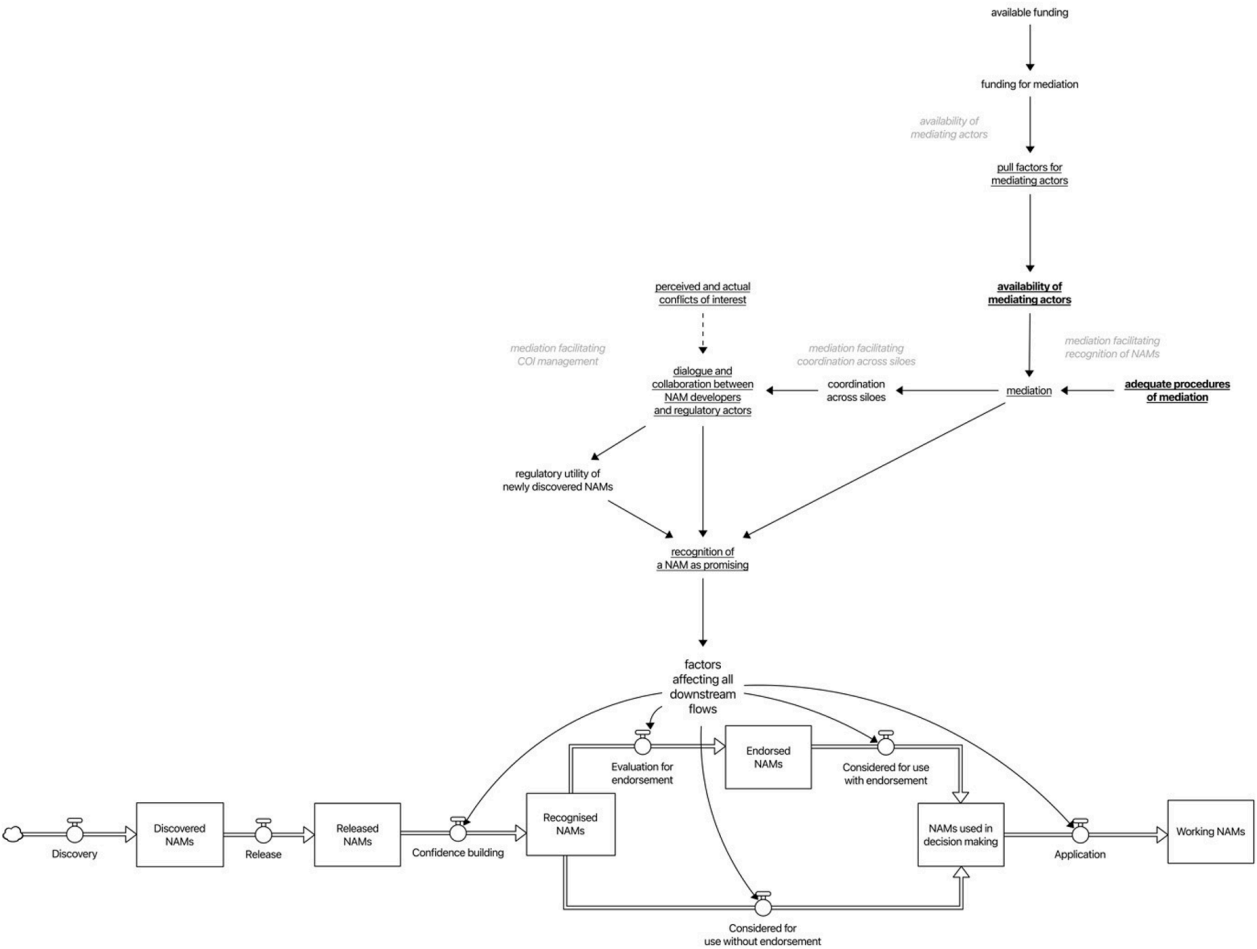
Section of the systems model that shows the “Mediation” mechanism.

**Fig. 6:**
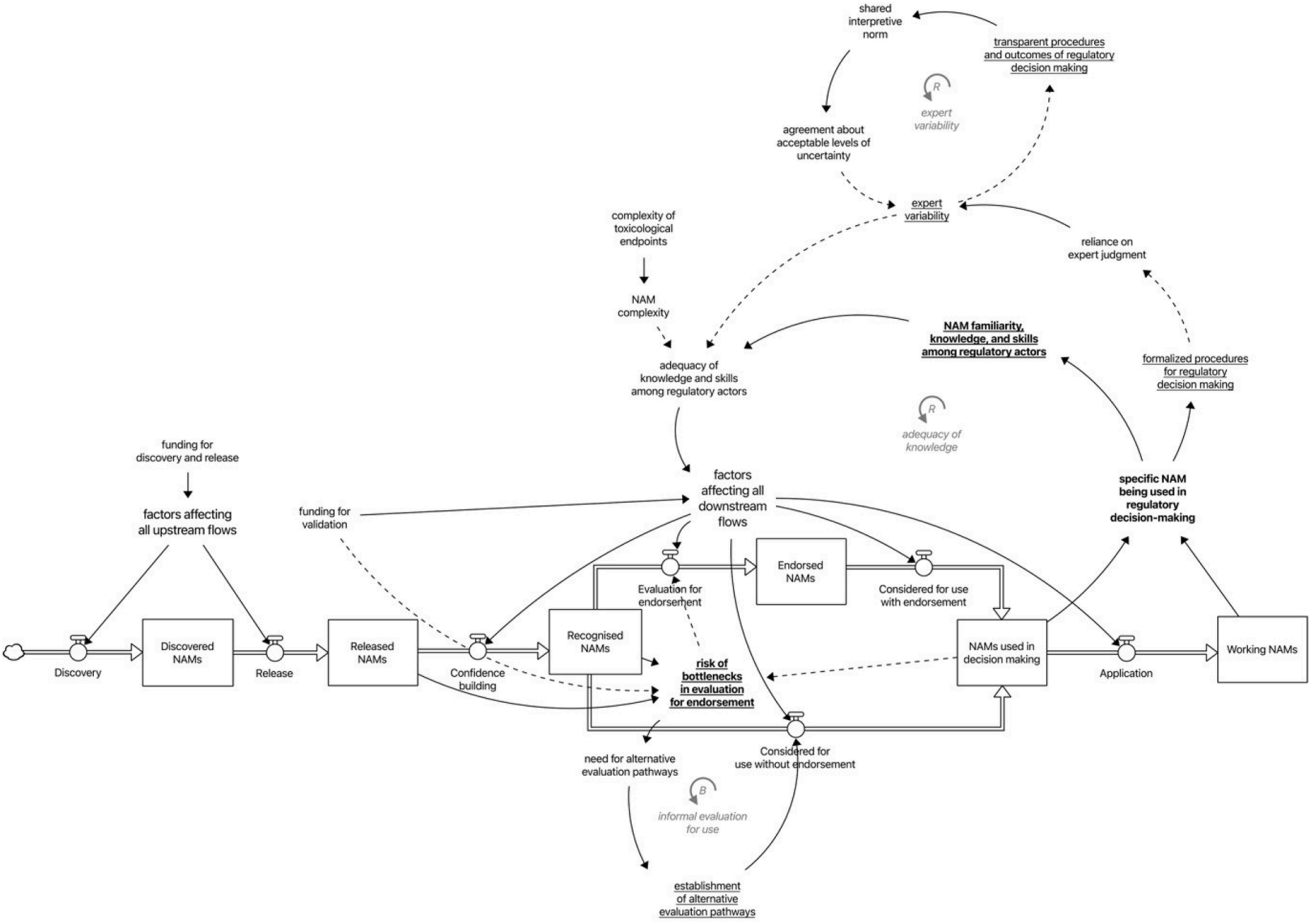
Section of the systems model that shows the “Innovation Outpacing Learning and Maturation” mechanism.

**Fig. 7:**
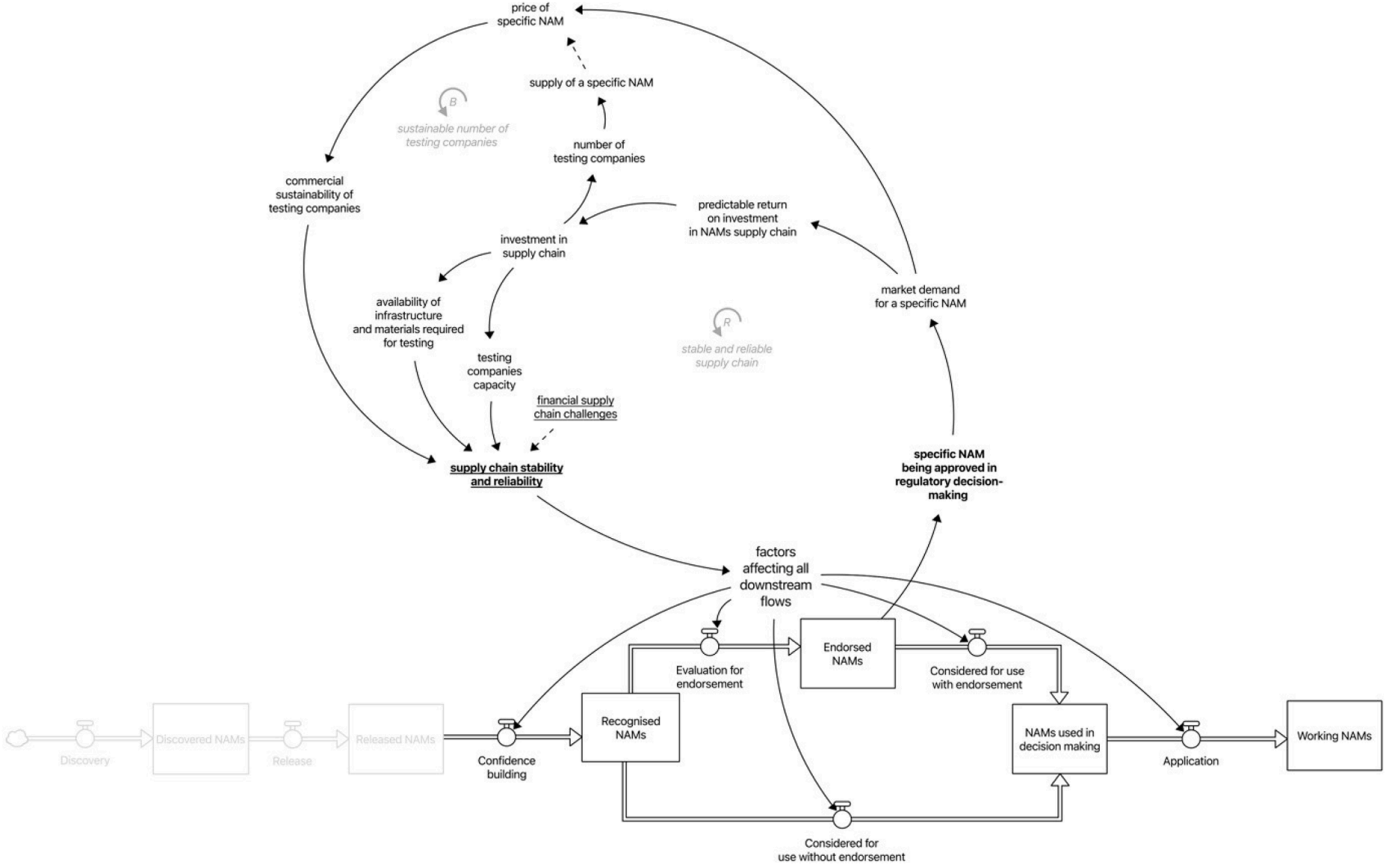
Section of the systems model that shows the “Supply Chain” mechanism.

**Fig. 8:**
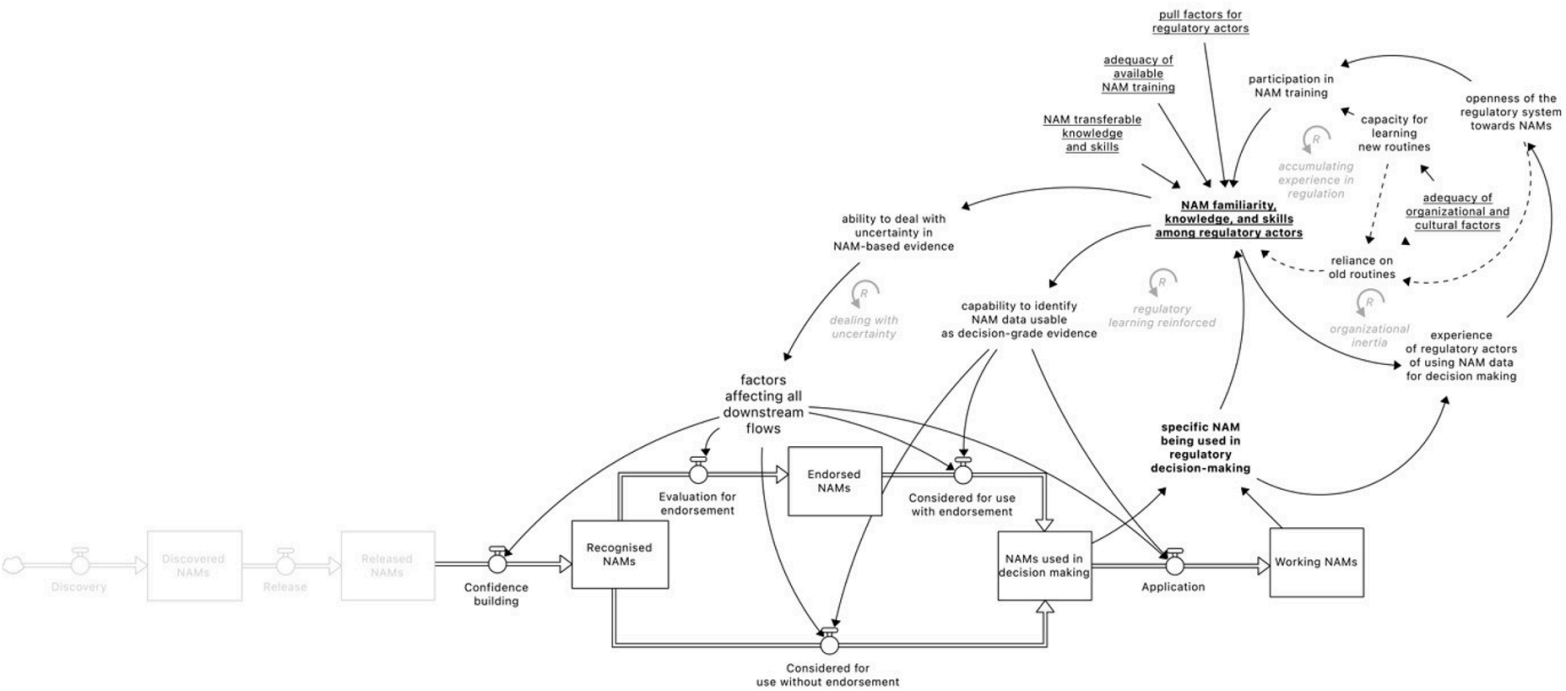
Section of the systems model that shows the “Regulatory Learning” mechanism.

**Fig. 9:**
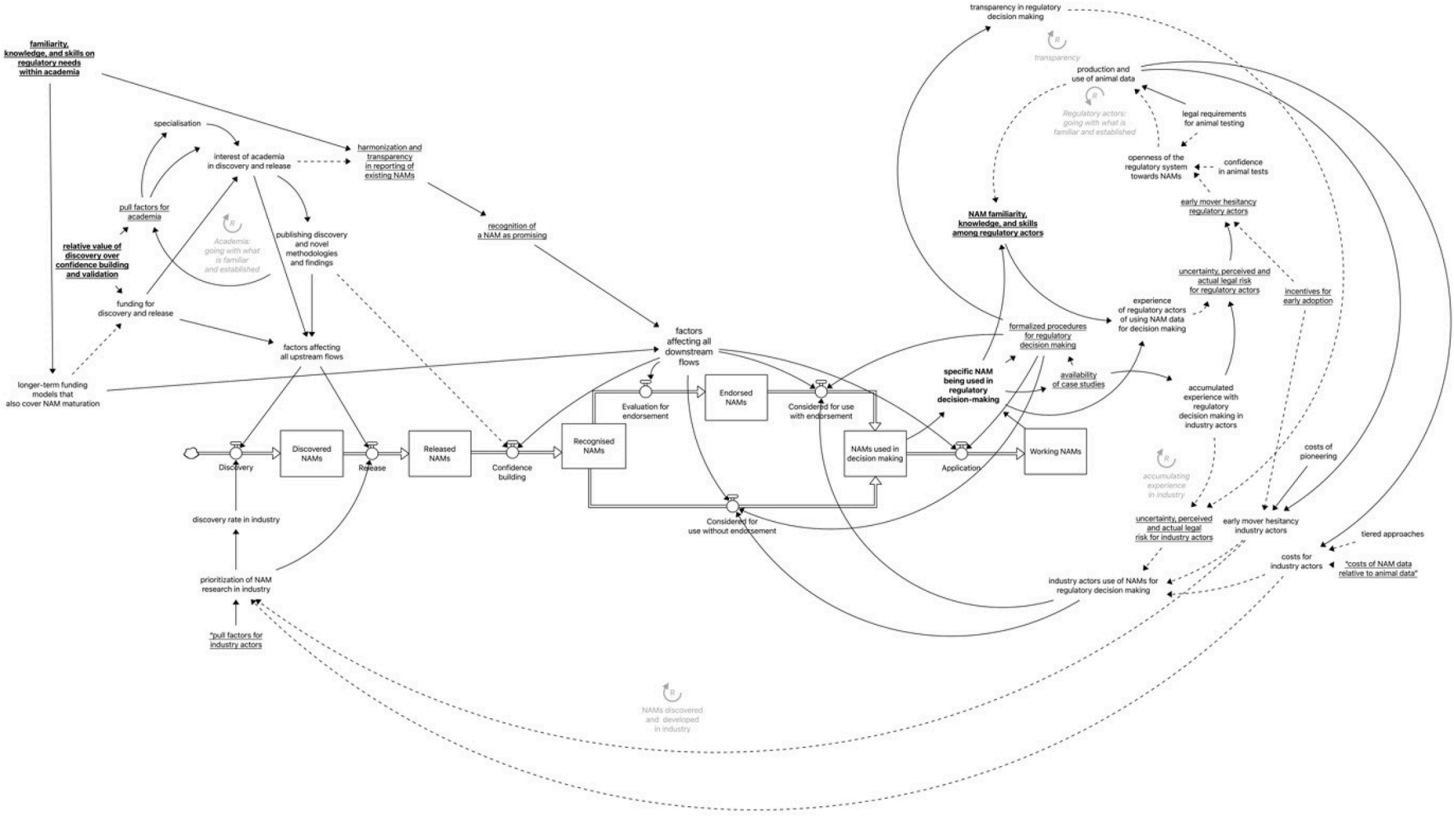
Section of the systems model that shows the “Going with What is Familiar and Established” mechanism.

The following sections present the overarching mechanisms and their feedback-loops from left to right along the NAM maturation chain, highlighting the actors involved at each stage. Fig. 4, Fig. 5, Fig. 6, Fig. 7, Fig. 8, Fig. 9 show each mechanism with their respective loops individually. Fig. A1 in the Appendix A illustrates how these mechanisms interact with each other and how the loops are all part of a larger whole.

**Table:** 

**A short primer on feedback loop diagrams in systems modelling** (Meadows, 2008; Sterman, 2000, 2002) Feedback loop diagrams are a tool for representing the feedback structure of a system. A feedback loop diagram consists of variables and arrows that denote the causal influences among the variables. Each causal link is assigned a polarity to indicate how the effect variable (dependent variable) changes when the cause variable (independent variable) changes. The conventions for drawing feedback loop diagrams in this article are that: • A solid arrow denotes that an increase in the cause variable leads to an increase in the effect variable t (and a decrease leads to a decrease). • A dashed arrow denotes that an increase in the cause variable leads to a decrease in the effect variable (and a decrease leads to an increase). An “R” as a loop identifier indicates a reinforcing loop, which means a feedback process in which a change in one direction leads to further changes in the same direction. This continuously amplifies, or rather, reinforces the original effect over time. A “B” indicates a balancing loop, which means a feedback process that counteracts change and promotes stability or equilibrium within a system.

#### 2.5.1 Mechanism: “Targeted research related to regulatory needs”

The “Targeted Research Related to Regulatory Needs” mechanism comprises two reinforcing loops, which describe the variables involved in establishing and communicating regulatory needs across siloes that then informs how effectively NAM research is targeted at those needs. This mechanism opens and closes all the downstream flows in the NAM maturation chain (i.e., flows starting from confidence building) and pertains primarily to NAM developers, researchers (within and outside academia, funders, publishers, and regulatory actors (see Fig. 4).

The reinforcing loop “Establishment and Consideration of Regulatory Needs” is rooted in the hypothesis that increased NAM familiarity, knowledge and skills (see Section 2.5.5. for a definition of these three concepts) among regulatory actors would enable regulatory actors to better characterise their needs regarding NAMs for regulatory decision making. This in turn would facilitate dialogue and collaboration between NAM developers from academia and industry and regulatory actors, which would positively impact the effective use of NAMs.

For instance, dialogue and collaborations with NAM developers and regulatory actors (e.g., in form of a transdisciplinary project) would help NAM developers understand regulatory needs, thereby increasing the regulatory utility of newly discovered NAMs and facilitating the recognition of a NAM as promising. A promising NAM has potential for informing decision making. Sufficiently harmonised and transparent reporting is a pre-requisite to determine whether it is indeed promising. Identifying and then prioritising “promising NAMs” for confidence building and evaluation for endorsement, is considered to contribute to effective use of NAMs by progression of potential relevant NAMs through the maturation chain. In turn, more specific NAMs would be used in regulatory decision making, which would increase the familiarity, knowledge and skills among regulatory actors and strengthen their capacity to characterise regulatory needs related to NAMs.

According to the “Validation Failure/Success” loop, perceived high failure rates in validation, meaning the – potentially unverified – expectation that validation of NAMs fails regularly, can reduce the available funding for validation. Reduced funding, in turn, increases the likelihood of actual validation failures (e.g., by missing out on the prevalidation step to reinforce the robustness of a NAM before entering formal validation), reinforcing perceptions of high failure rates and creating a negative feedback loop. A more systematic approach to identifying and then prioritising promising NAMs for e.g., pre-validation could potentially interrupt this cycle by lowering actual failure rates of validation, improving perceptions of validation success, and thereby increasing the availability of funding for validation.

#### 2.5.2 Mechanism: “Mediation”

The “Mediation” mechanism describes how siloed actors in the system can be connected by *mediators*, a type of actor that takes on an intermediary role between disconnected actors (in our model, for example between NAM developers from academia or industry and regulatory actors) to facilitate the maturation of NAMs.

The “Mediation” mechanism opens and closes the confidence building, evaluation for endorsement, considered for use with and without endorsement and application flows in the NAM maturation chain (see Fig. 5). Mediators play a key role in facilitating dialogue and collaboration and managing conflicts of interest (COI) in the development and uptake of NAMs (“Mediation Facilitating COI Management”). When mediating actors and adequate mediation procedures are available, mediation can strengthen COI management and promote dialogue and collaboration between NAM developers, researchers and other stakeholders. This collaboration improves the regulatory utility of newly developed NAMs and supports the recognition of promising NAMs (“Mediation Facilitating Recognition of NAMs”). Mediation also helps bridge disciplinary and institutional siloes (“Mediation Facilitating Coordination Across Siloes”), further enhancing cooperation and the identification of promising NAMs, which in turn supports all downstream processes. The availability of mediating actors depends on sufficient pull factors, including dedicated funding for mediation and other incentives for mediation activities (“Availability of Mediating Actors”).

#### 2.5.3 Mechanism: “Innovation outpacing learning and maturation”

The “Innovation Outpacing Learning and Maturation” mechanism comprises three feedback loops and describes how currently the fast pace of NAM innovation outpaces regulatory processes and system learning. The mechanism focuses primarily on NAM discovery and thus, on NAM developers (e.g., academia, industry) (see Fig. 6). The high number of NAMs being released relative to the capacity to evaluate them for endorsement and considering them for use creates a bottleneck between innovation and application. Increased funding for discovery and release can lead to a larger pool of released NAMs, but if evaluation does not keep pace, a bottleneck can appear which prevents NAMs that may be promising from being evaluated, endorsed and used. Limits on regulatory evaluation include lack in funding or skilled personnel or time pressure, whereas limits on evaluation may include lack of documentation that would support evaluation or NAMs being designed in such a way that their potential application may be unclear.

The “Adequacy of knowledge” loop states that consideration for use depends in part on regulatory actors’ familiarity, knowledge and skills regarding NAMs, which are shaped by the adequacy of available training, the availability of qualified professionals, and the complexity of toxicological endpoints and NAMs. Validation of NAMs for their intended fit-for-purpose use also contributes to this regulatory uptake of NAMs. While greater regulatory competence supports the evaluation and application of NAMs, it can also increase pressure on downstream flows if many NAMs enter the pipeline simultaneously.

According to the “Informal evaluation for use” loop, alternative evaluation pathways that facilitate consideration for use without endorsement and increase the number of NAMs used in decision making, can alleviate the bottleneck. Informal evaluation is any process in which data from a NAM is evaluated whether it is fit for decision-making. Examples of such informal evaluation may include in-house testing of a NAM; the use of systematic review or other evidence synthesis and mapping methods; or the use of expert elicitation. The specific purpose will vary depending on the involved actor but would concern any decision for which an endorsed NAM would not be required to be used. Recognition of promising NAMs, supported by greater harmonisation and transparency in reporting, can further encourage the establishment of such pathways.

According to the “Expert variability” loop, the alternative evaluation pathway and the absence of formalised procedures for regulatory decision making applying NAMs might place more emphasis on expert judgment, meaning experts from various disciplines relevant to the NAM at hand. A higher emphasis on expert judgment enables the use of NAMs as a versatile tool for decision making. However, higher reliance on expert judgment can be vulnerable to expert variability, particularly in the absence of formalised decision making guidance, which in turn might negatively influence the transparency of procedures and outcomes of regulatory decision making. This in turn reduces the shared interpretative norm and agreement about acceptable levels of uncertainty, which again increases expert variability.

#### 2.5.4 Mechanism: “Supply chain”

Fig. 7 displays the “Supply Chain” mechanisms and its two loops. This mechanism’s premise is that to increase the flows of NAMs through confidence building, evaluation for endorsement, consideration for use with and without endorsement, and application, a stable and reliable supply chain is needed (“Stable and Reliable Supply Chain” loop). This mechanism opens and closes in particular the downstream flows in the NAM maturation chain and pertains primarily to regulatory actors and supply chain actors (e.g., Contract Research Organisations (CROs), in-house laboratories in industry, cell banks). The mechanism describes that to approve and use NAMs for regulatory decision making, a stable and reliable supply chain is needed, which might not develop in the absence of regulatory approval and use. Various technical and financial supply chain challenges listed in Table 1 disrupt the reliability and stability of the supply chain. A stable and reliable supply chain for a specific NAM can only form if there is a market demand for it and a predictable return-on-investment, which leads to investment in the supply chain. Market demand substantially increases for NAMs that have been approved or are regularly used in regulatory decision making.

**Table 1:** Selected technical and financial supply chain challenges of a stable and reliable NAM supply chain.

Technical supply chain challenges	Financial supply chain challenges
• Availability of resources or infrastructure • Availability of essential NAM components	• Limited funds in chemical industry due to global competition • High regulatory costs • Energy price pressures and economic crises
• Complexity of NAMs	• Monopolies in key resources (e.g., critical cell lines, cell cultures)
• CROs capacity and (prospective) testing volume	• High cost of laboratory equipment and consumables for some NAMs
	• Lack of staff with required expertise
	• Intellectual property concerns over NAMs
	• Lack of commercialisation strategies for NAMs

Stability comes from a market that features an optimal number of diverse, financially stable testing facilities with capacity for NAM testing (“Sustainable number of testing companies” loop). Too few CROs offering a particular NAM leads to high costs and slow turnaround, while too many CROs lead to low prices and commercial unsustainability of assays. Reliability concerns the commercial availability of infrastructure and materials required for testing (i.e., NAMs components with proper quality and characterisation, data sharing or analysis platforms), the availability of qualified staff and regularly updated protocols.

#### 2.5.5 Mechanism: “Regulatory learning”

The Regulatory Learning Mechanism features four loops, displayed in Fig. 8. This mechanism primarily pertains to the downstream flows and centres around regulatory actors, while also potentially including other actors. The loops in this mechanism focus on the availability and distribution of the following types of learning among regulatory actors:

• **Familiarity with NAMs**, which denotes the extent to which NAM-related terminology, concepts, and methodologies are cognitively accessible and recognisable to an individual.
• **Knowledge of NAMs**, which refers to the extent to which an individual possesses accurate, retrievable information about the principles, methodologies, applications, and limitations of NAMs.
• **Skills in NAMs,** which is an individual’s demonstrated ability to apply NAMs effectively in practice, including selecting appropriate methodologies, executing or interpreting NAM-based studies, and integrating their outputs into regulatory or scientific decision making.

The use of a specific NAM in regulatory decision making increases familiarity, knowledge and skills among those regulatory actors that require it. This strengthens the regulatory actors’ ability to recognise NAM data as decision informing evidence (e.g., through institutional endorsement, systematic reviews, expert elicitation), handle uncertainty in NAM-based evidence, and consider NAMs for use with or without endorsement, ultimately increasing their effective application (the “Dealing with Uncertainty” loop). Growing experience with NAM data further promotes openness within regulatory systems and reduces reliance on established routines, creating a reinforcing learning cycle (the “Regulatory learning reinforced” loop).

Organisational and cultural factors can hinder this learning process by limiting the capacity to adopt new practices and maintaining reliance on existing routines (the “Organizational inertia” loop). Organisational structures hamper learning if they feature rigid hierarchies and managerial siloes, lack incentives, resources and capacity for training due to competing priorities and time pressure, or do not possess infrastructure and processes for the systematic storing and sharing of data. Cultural factors that challenge learning are primarily organisational inertia and habit, work routines with little time or flexibility, and a mismatch between (perceived) information needs and learning formats. Pull factors for learning, adequate and targeted NAM training, and the development of transferable knowledge and skills (e.g., legal pre-requisites, science communication) can strengthen regulatory competence with NAMs, thereby supporting confidence building, evaluation for endorsement, and broader regulatory uptake (“Accumulating experience in regulation” loop).

Adequate and targeted NAM training adjusts the level of the learning to the individual’s information needs. For instance, a high-level decision maker (e.g., risk manager, group or team leader) does not have the capacity or need to develop in-depth knowledge and skills related to NAMs but needs to be familiar with terminology, concepts and methodologies, as well as potential limitations. On the other hand, risk assessors require NAM-specific knowledge and skills to apply them. Reasons for a mismatch between what learning opportunities are offered and which ones are needed may be that trainers focus on the parts of the NAM chain that comes most naturally to them (i.e., discovery and implementation, rather than translation, evaluation and integration in regulatory processes).

#### 2.5.6 Mechanism: “Going with what is familiar and established”

The “Going with What is Familiar and Established Mechanism” with its five loops (see Fig. 9) is closely related to the Regulatory Learning Mechanism in that it also focuses on the familiarity, knowledge and skills of actors within the system. As this increases through the use of specific NAMs in regulatory decision making, it reduces uncertainty and perceived legal risks for regulatory and industry actors, lowers first-mover hesitancy across the system, and increases the openness towards NAMs, thereby gradually reducing reliance on animal data (“Regulatory actors: Going with what is familiar and established” loop). However, strong confidence in animal tests and legal requirements for animal testing reflected in current regulatory structures (e.g., standard information requirements within REACH), hinder this gradual shift. Incentives for early adoption and reduced pioneering costs can mitigate first-mover hesitancy among both regulatory and industry actors.

As regulatory openness increases and animal data use declines, costs and risks for industry decrease, encouraging greater prioritisation of NAM research and supporting both the discovery and release of NAMs (“NAMs discovered and developed in industry” loop). The use of NAMs in regulatory decisions ideally also generates case studies and contributes to the development of procedures for more formalised and transparent regulatory procedures (“Transparency” loop). Greater transparency further reduces (legal) uncertainty for industry, increasing the use and consideration of NAMs for example in regulatory submissions (“Accumulating Experience in Industry” loop).

At the research level, stronger incentives and funding for discovery could stimulate academic engagement in developing new NAMs (“Academia: Going with what is familiar and established” loop). However, a strong focus on discovery alone may reduce harmonisation and transparency in reporting existing NAMs. Greater familiarity within academia with regulatory needs along with longer-term funding models that support NAM maturation, could improve regulatory utility and reporting standards.

## 3 Discussion

### 3.1 Summary and discussion of results

The diverse data collection methods applied in this study have generated a rich and thick data set of mechanisms, processes and loops that currently hinder or might in the future drive the effective use of NAMs. This system analysis highlights that the uptake of NAMs in regulatory decision making is shaped by a complex set of interacting factors that go beyond technical opportunities and limitations of NAMs. While these are important and included in the loops at various points, in this article we want to call attention to system factors that are difficult to observe.

An overall pattern that emerged is that most causal feedback loops in the systems model are reinforcing loops, with only a few balancing loops. Reinforcing loops amplify change over time, while balancing loops counteract change and stabilise the system. The loops primarily loosen upstream flows (such as discovery, release, and confidence building), – while constricting downstream flows (evaluation for endorsement, consideration for use with and without endorsement, and application). These patterns explain why NAM uptake in regulatory toxicology is inefficient. Most mechanisms reinforce the use of animal testing over time, and work against the effective use of NAMs, primarily constricting downstream flows but not upstream flows. A wealth of NAMs is being discovered and released, while only few mature into useful tools for regulatory decision making.

With the goal to bring forward the date of the effective use of NAMs, our study also identified potential leverage points that could speed up this transition of vicious cycles into virtuous ones. Some of these leverage points are apparent and have already been pointed out in the literature, among them the need to intensify NAM training for regulatory actors, the promotion of dialogue and collaboration between NAM developers and regulatory actors or ensuring a stable and reliable supply chain (Bearth, Kopainsky et al., 2025, 2024; Berggren and Worth, 2026; Meigs et al., 2018). Notably, the economic dimensions, including the costs of animal testing, the economic consequences of false-positive and false-negative test results, and the emerging business opportunities in alternative methodologies, have been summarised in a series of recent publications (Bottini, 2009; Meigs et al., 2018).

To translate these potential leverage points into interventions, an additional analysis step is needed that requires intervention design. Intervention design is the deliberate process of developing specific actions that aim to change the system’s behaviour. System change is tackled by targeting specific underlying structures, reversing loops from vicious to virtuous, replacing infrastructure and processes with new ones, introducing opportunities or inhibiting barriers within the system. Intervention design will be the goal of the third and final phase of CHANGE; subsequently, some leverage points for intervention are identified based on our systems model.

### 3.2 Recommendations for leverage points for intervention based on central dynamics

Our systems model of the regulatory toxicology system from the perspectives and experiences of actors operating within allows us to foresee roadblocks, facilitating or inhibiting conditions and connections with other mechanisms that would likely enhance the effectiveness of interventions. A central dynamic concerns the fact that all actors in the system, operating within their siloes, defined responsibilities, and incentive structures, are prone to stick to what is familiar and established. Many causal feedback loops require overcoming these dynamics by several actors simultaneously. This can lead to a “Catch-22,” in which a) NAMs need to be used to make actors more familiar with them and establish them as decision-grade evidence and b) NAMs are not used because actors are unfamiliar and they’re not established. While this – admittedly – seems difficult to overcome, our system model shows that adequate organisational and cultural factors, as well as adequate training, and installing pull factors play a central role here.

First, according to our data, adequate organisational and cultural factors are ensured through shallow hierarchies that allow for input from early- and mid-career skilled experts, coordination across institutional siloes, minimal financial, time and workload constraints, expertise in quantitative and computational science, proprietary data systems, and regulatory openness towards NAM use.

Second, adequate training allows regulatory actors to attend the type of training from which they would benefit the most. For example, when developing a NAM training for regulatory actors that features theoretical information about a specific NAM and/or some hands-on training in using the NAM in regulatory decision making several things should be considered. The training should provide sufficient information on the NAM so that regulatory actors can assess when it is appropriate to use it (i.e., appropriateness of NAM for specific regulatory questions) and whether it has been conducted and reported to an adequate standard, thereby strengthening their capability to identify NAMs as decision informing evidence (i.e., quality and reliability of the underlying data, uncertainty assessment). This would gradually change the vicious loop of regulatory actors going with what is familiar and established through the increasing accumulation of NAM experiences in regulation, ability to deal with uncertainty, and transparency. To support the mechanisms further, the training should include transferable knowledge and skills needed for regulatory decision making, such as relevant legal and regulatory requirements. It should offer hands-on training in interpreting and applying the NAM data in regulatory decisions, recognising that a single training event may be insufficient. When planning the training, the participants’ incentives for participation and their capacity for learning new routines should be assessed and addressed, as organisational and cultural factors in their institutions may hamper translating the learnings into action (e.g., in form of hands-on guides on how to address or overcome these barriers). Another important question regarding training is which actors are best equipped to deliver such a training for regulatory actors? Trainers would need to bring along experience in NAMs but also awareness for their audiences’ pre-conditions, needs and restrictions, which is a tall order for early- and mid-career researchers working for example within large-scale projects on NAM development and validation. This highlights the need for more experienced trainers, perhaps with experience across regulatory siloes, to not just provide the necessary familiarity, knowledge and skills to regulatory actors, but to translate technical evidence into use for regulatory use.

Third, pull factors are conditions, incentives and opportunities that pull actors towards contributing in a meaningful way to the NAM maturation chain. Acknowledging differences and similarities across actors can be a first step towards installing more relevant pull factors for the effective use of NAMs.

• For academia, these are pull factors towards engaging in the discovery and release of NAMs, such as opportunities for publication, access to funding, and possibilities to develop new methodologies and generate knowledge.
• For industry actors, these are pull factors towards prioritising NAM research, such as a potential competitive advantage in the market, expected returns on investment, technological innovation needs, commercial gains, recognition, regulatory alignment, and opportunities for learning and growth.
• For regulatory actors, these are pull factors towards engaging with NAMs, such as availability of additional time, motivational or financial resources, and opportunities to gain experience.
• Lastly, for mediating actors, these are pull factors towards engaging in facilitation and translation activities.

Returning to the latter point in more detail, the systems model highlights that the middle section of the NAM maturation chain is the most challenging part of the system to influence due to the need for dialogue and collaboration but lacking sufficient mediating actors between academia, regulatory and industry actors. The call for independent mediators that facilitate dialogue and collaboration, ensure adequate conflict of interest management and take on the challenging task to recognise NAMs as promising and prioritising their maturation appeared frequently in the data. Less frequent, and potentially telling of a deeper-seated issue, were concrete examples of pull factors for such mediating actors, which are particularly important considering the regulatory and scientific skills these mediating actors would need to bring to the table. Thus, determining who these mediating actors could be, and which infrastructure and processes are needed to facilitate mediation is a core opportunity within the field.

The systems model also identifies the potential for downstream bottlenecks when large numbers of NAMs are developed and released but regulatory evaluation, recognition and decision making capacity does not expand accordingly. Such bottlenecks may stimulate the development of alternative evaluation pathways for recognised NAMs that allow methods to inform regulatory decisions without full formal endorsement (e.g., systematic review, expert elicitation). According to our workshop participants and the system model, recognition of promising NAMs, supported by harmonised and transparent reporting, could potentially help prioritise methods and facilitate these processes, not least the validation of NAMs for their intended fit-for-purpose use. Again, this refers back to the need to introduce new actors, specifically taking on mediation and the middle section of the NAM maturation chain, holds great potential here. For example, a “NAM triage organisation” with a good understanding of regulatory need, tasked with evaluating existing NAMs and prioritising some for validation and endorsement, could alleviate the pressure on the bottleneck.

Challenges emerge particularly for the middle and downstream sections of the NAM maturation chain, specifically in confidence building, evaluation for endorsement, and consideration for use with and without endorsement. Several system-level factors further shape the adoption of NAMs in regulatory toxicology. One concerns the validation process: when validation is perceived as likely to fail, funding for validation decreases, which can in turn increase actual failure rates and reinforce negative perceptions. Recognition of a NAM as promising can break this cycle by improving validation outcomes and restoring confidence among funders. Another dynamic involves the supply chain and market structure for NAMs. Regulatory endorsement and use create market demand and predictable returns on investment, which encourage investment in infrastructure, materials and testing capacity, as companies increasingly rely on CROs for animal testing and NAMs (Meigs et al., 2018). The in vitro toxicology testing market has been valued at several billion US dollars with compound annual growth rates exceeding 6%, reflecting a shift in the commercial landscape that could accelerate or impede NAM supply chain development depending on how these market forces are channelled (Meigs et al., 2018). Our systems model, rooted in the empirical data from workshop 1 and 2, suggests that if increased supply reduces prices excessively, the commercial sustainability of testing providers may be undermined, potentially threatening supply chain stability.

Finally, the systems model highlights the importance of incentive structures across sectors. Greater transparency in regulatory procedures, and the publication of case studies demonstrating NAM use can lower perceived legal and financial risks for industry. Framing the NAM transition in economic terms – as an investment with positive returns – may resonate with industry decision makers and policymakers who are less motivated by ethical or scientific arguments alone. This could encourage greater use of NAMs in regulatory submissions and increases industry investment in NAM research and development. At the same time, academic incentives strongly influence the balance between discovery of new methods and the maturation, validation and harmonisation of existing ones. Long-term funding models that support the full maturation pathway of NAMs can improve regulatory utility, although they may reduce resources available for early-stage discovery.

### 3.3 Strengths and limitations of the methodological approaches

The qualitative data collection, analysis and interpretation used in this study present both strengths and limitations. The qualitative approach to data collection allowed for the in-depth exploration of participants experiences, relying on their stories and system knowledge. The project team were reflexive during the planning, conducting and analysis of the data, reflecting on their own roles and knowledge of the system and how this could impact their interpretations. The selection of participants can influence the depth of the collected data and thus represents a potential limitation. To address this, we sought to include a heterogeneous and international group of participants with recent or extensive experience in the regulatory toxicology system. Follow-up workshops were further used to compensate for the under-representation of certain participant groups. Nevertheless, some sectors and regions were overrepresented in our participants (e.g., Europe, USA) and others underrepresented (Africa, South America, Asia; cf. Appendix B for an overview of sectors and geographical regions of the participants), or the analysis of the data might have been impacted by repeated, strong opinions voiced in the workshops. The use of input presentations to stimulate discussion likely influenced the types of examples and experiences shared by participants. Jones et al. (2025) describe several of the approaches employed to encourage open dialogue and active exchange during these sessions. The analysis of the extensive dataset also posed challenges. These were partly addressed through the involvement of an interdisciplinary project team that met frequently over a two-year period to jointly review and reflect on the analysis. In addition, each month progress was presented to and discussed with an advisory board. As the system model was derived from participants’ accounts and interpretations, it reflects perceived rather than empirically verified relationships. The aggregation of perspectives into a single diagram may obscure disagreements and heterogeneity among participants. The value of the model lies in how it supports the design of system-level interventions, by providing a means to comprehensively identify and reflect upon system factors, while grounding such reflection in a theoretical framework that shows how potential interventions can be expected to work. Overall, the model should not be viewed as complete or strictly correct, but judged on whether it is useful for the promotion of the effective use of NAMs.

## 4 Conclusion

Utilising the systems thinking approach adopted in this article, regulatory toxicology can be understood as a complex evolving machine that has been assembled over time through the gradual addition of levers, gears and mechanisms in response to scientific advances and regulatory needs. Within this machine, NAMs represent new components that have the potential to improve the machine’s performance, but their effective integration depends on how well they interact with the existing mechanisms. Successful system change requires more than simply inserting new technical parts into the system (Ulnicane, 2015). The functioning of the machine also depends on supporting mechanisms; in our systems model those are regulatory expertise, validation processes, funding structures, supply chains, and collaboration among contributors, that allow these new components to operate smoothly. If these elements are misaligned or fragmented, friction and bottlenecks may arise, slowing the system or preventing promising methods from moving through it.

From a systems perspective, accelerating the effective uptake of NAMs therefore requires adjustments across the machine. Strengthening key leverage points, such as regulatory capacity, transparency in decision making, mediation between actors, and sustained support for method maturation, can help ensure that new components are not only added to the system, but are effectively connected to the gears that drive regulatory decision making. By gradually recalibrating the machine in this way, regulatory toxicology can evolve to incorporate innovative methodologies while continuing to fulfil its core objective of protecting human health and the environment. We hope that our novel perspective, utilising systems thinking and considering the perspectives and experiences from diverse actors in the field contributes to this goal.

## 5 Disclaimer

The views and opinions expressed by the authors are their own and do not necessarily represent the views or policies of their employers or organizations. EFSA is not responsible for any use that may be made of the information it contains.

## Ethics declaration

### Informed consent and patient details

Written informed consent to take part in the study and to publish the article has been obtained from all participants or their legal representatives. The privacy rights of participants have been observed.

### Studies in human

This study was performed in compliance with relevant laws, regulatory frameworks and guidelines where the research took place.

Ethics committee approval was not required under relevant laws and institutional guidelines. The workshop does not involve the collection or processing of personal data and is therefore exempt from requiring a data protection impact assessment (DPIA) according to the Norwegian legislation. Since no biological materials or information on health will be collected and/or processed in the workshop, the workshop is also exempt from requiring ethics approval. However, due to the nature of the discussion themes during the workshop, we have assessed and approved measures to increase the confidentiality and anonymisation of the raw data collected (i.e., secure storage on access-controlled/password-protected servers, pseudonymisation, informed consent)

## CRediT authorship contribution statement

**Angela Bearth:** Writing – review & editing, Writing – original draft, Visualization, Methodology, Investigation, Formal analysis, Conceptualization. **Birgit Kopainsky:** Writing – review & editing, Writing – original draft, Visualization, Methodology, Investigation, Formal analysis, Conceptualization. **Lowenna B. Jones:** Writing – review & editing, Methodology, Investigation, Conceptualization. **Gunn E. Vist:** Writing – review & editing, Methodology, Investigation, Funding acquisition, Conceptualization. **Trine Husøy:** Writing – review & editing, Methodology, Investigation, Funding acquisition, Conceptualization. **Camilla Svendsen:** Writing – review & editing, Methodology, Investigation, Funding acquisition, Conceptualization. **Paul Whaley:** Writing – review & editing, Methodology, Investigation, Funding acquisition, Conceptualization. **Sebastian Hoffmann:** Writing – review & editing, Methodology, Investigation, Funding acquisition, Conceptualization. **Heather M.R. Ames:** Writing – review & editing, Methodology, Investigation, Funding acquisition, Conceptualization. **Denise Bloch:** Writing – review & editing, Methodology. **Aleksandra Čavoški:** Writing – review & editing, Methodology. **Holly G. Davies:** Writing – review & editing, Methodology. **Arianna Giusti:** Writing – review & editing, Methodology. **Thomas Hartung:** Writing – review & editing, Methodology. **Seok Kwon:** Writing – review & editing, Methodology. **Olivia J. Osborne:** Writing – review & editing, Methodology. **Christophe Rousselle:** Writing – review & editing, Methodology. **Jennifer B. Sass:** Writing – review & editing, Methodology. **Ulla Simanainen:** Writing – review & editing, Methodology. **Kristina A. Thayer:** Writing – review & editing, Methodology. **Daniele Wikoff:** Writing – review & editing, Methodology. **Gro H. Mathisen:** Writing – review & editing, Project administration, Methodology, Investigation, Funding acquisition, Formal analysis, Conceptualization.

## Declaration of competing interest

The authors declare the following financial interests/personal relationships which may be considered as potential competing interests: Gro H. Mathisen reports financial support was provided by European Food Safety Authority. Gro H. Mathisen reports financial support was provided by Norwegian Scientific Committee for Food Safety. Gro H. Mathisen reports financial support was provided by Evidence-Based Toxicology Collaboration. The authors have no conflict of interest. Several of the co-authors are currently working on one or more of the EU funded projects ONTOX (Grant 963,845), NAMWISE (Grant 101,191,595), and PARC (Grant 101,057,014). PW provides consultancy services to the Evidence-Based Toxicology Collaboration, in which EFSA as a funder of CHANGE is a Member of the Board of Trustees. The author Kristina A. Thayer is employed at California Environmental Protection Agency’s Office of Environmental Health Hazard Assessment (OEHHA). The views are those of the author and do not necessarily reflect the views or policies of OEHHA or California Environmental Protection Agency. The views and opinions expressed by the authors are their own and do not necessarily represent the views or policies of their employers or organizations. EFSA is not responsible for any use that may be made of the information it contains. If there are other authors, they declare that they have no known competing financial interests or personal relationships that could have appeared to influence the work reported in this paper.

## Figures and Tables

**Table B1:** Overview of participants in workshops 2024 and 2025.

	Workshops 2024 (incl. follow-up online)	Workshop 2025
**Sector**^1^		
Academia (e.g., university, research institute)	25	16
Consultancy and/or freelance	2	4
Contract Research Organization (CRO)	0	1
Industry (e.g., business, company, trade)	13	8
International authority or agency (e.g., EFSA, ECHA)	5	3
International scientific organisations and/or committees (e.g., WHO, OECD)	4	4
National authority or agency	25	19
Non-governmental organisations (NGOs) and/or non-profit organisations (NPOs)	5	5
**Region**		
**Europe**	**49**	**34**
Austria	1	1
Belgium	6	1
Bulgaria	1	0
Denmark	1	1
Estonia	1	0
Finland	1	1
France	4	2
Germany	2	3
Italy	3	3
Latvia	1	0
Malta	1	1
Norway	12	12
Spain	1	1
Switzerland	4	2
The Netherlands	4	5
United Kingdom	6	1
**North America**	**14**	**11**
United States of America	14	11
**South America**	**2**	**0**
Brazil	2	0
**Asia**	**5**	**2**
China	1	0
Singapore	3	1
Taiwan	1	1
**Australia**	**1**	**0**
Australia	1	0

1 Participants were invited to indicate everything that applied.

## Data Availability

The data that has been used is confidential.

## Appendix C Supplementary materials

Image, application 1
