# Supplementary material for: Modelling the regulatory toxicology system to promote the effective use of new approach methodologies: a system thinking approach
Source: NAM J. 2026 Jul 24;2:100120. doi: 10.1016/j.namjnl.2026.100120 (PMC13450631; doi:10.1016/j.namjnl.2026.100120)
Supplement: Supplementary file 1 [file mmc1.docx]

# Workbook – Theme 1

# Making NAMs accepted and accessible

The workbook contains 14 hypotheses derived from the dataset collected in the first phase of CHANGE.

We have developed this workbook to collect your individual feedback on these hypotheses, and to refine our initial theories about how the regulatory toxicology system functions around NAMs.

*Content of the workbook:*

- An overview of the 14 hypotheses (three top-level hypotheses with sub-hypotheses).
- Each hypothesis presented separately with a 5-point Likert scale for rating your agreement with the hypothesis and space to include comments or visualisations specific to the hypothesis.
- A blank page at the end for other comments/visualisations.

**Overview of the 14 hypotheses**

1. **The legal, financial, and perceived risks of using NAMs as evidence in regulatory toxicology means the system defaults to animal data.**
   1.1. The legal realities of using data in decision-making defeats the aspiration to move towards NAMs and away from tried-and-true animal data.
   1.2. Perceived legal risks of using NAMs for making regulatory decisions are based on bad previous experiences and relentless criticism, which drives the perception among regulators that NAMs are not worth the risk.
   1.3. There is nothing to be gained from being the first individual or institution to use NAMs in regulatory decision-making.
2. **There exist substantial finance and supply chain issues inhibiting the NAM discovery to validation process, which are currently overlooked.**
   2.1. Supply chain infrastructure (e.g. availability of cell material, data storage facilities, etc.) is too unpredictable and fragmented to support scaling-up of the use of NAMs.
   2.2. Moving NAMs forward from discovery to validation requires specific competencies and quality assurance processes (e.g., certified laboratories) that are too inaccessible to most actors involved in NAM development.
   2.3. Financial issues in the supply chain (e.g., requirement of expensive testing equipment) are prohibiting the scaling-up of the use of NAMs.
   2.4. The economics of the supply chain are insufficiently anticipated and managed when popularising NAMs.
   2.5. The relative costs of animal testing, in comparison to the relative costs of NAMs, disincentivises NAM use.
3. **Funding is structured in a way that does not support the popularisation and validation of promising NAMs.**
   3.1. Discovery is more attractive and comes more naturally to funders than popularisation and validation (e.g., easier to fund, asked for by their constituents, easier to find cost-effective labour to do the work, output is more measurable, higher quantity of projects per unit cost).
   3.2. Popularisation and validation of NAMs requires continuous and uninterrupted funding, which is hard for funders to provide (e.g. expense, monitoring).
   3.3. Funding cycles are too short or out of step with current regulatory priorities for popularisation and validation of NAMs that meet regulators’ needs.
1. **The legal, financial, and perceived risks of using NAMs as evidence in regulatory toxicology means the system defaults to animal data.**

| Strongly disagree | Disagree | Agree | Strongly agree | Do not know |
| --- | --- | --- | --- | --- |
|  |  |  |  |  |

Comments:

a. The legal realities of using data in decision-making defeat the aspiration to move towards NAMs and away from tried-and-true animal data.

| Strongly disagree | Disagree | Agree | Strongly agree | Do not know |
| --- | --- | --- | --- | --- |
|  |  |  |  |  |

Comments:

b. Perceived legal risks of using NAMs for making regulatory decisions are based on bad previous experiences and relentless criticism, which drives the perception among regulators that NAMs are not worth the risk.

| Strongly disagree | Disagree | Agree | Strongly agree | Do not know |
| --- | --- | --- | --- | --- |
|  |  |  |  |  |

Comments:

c. There is nothing to be gained from being the first individual or institution to use NAMs in regulatory decision-making.

| Strongly disagree | Disagree | Agree | Strongly agree | Do not know |
| --- | --- | --- | --- | --- |
|  |  |  |  |  |

Comments:

2. **There exist substantial finance and supply chain issues inhibiting the NAM discovery to validation process, which are currently overlooked.**

| Strongly disagree | Disagree | Agree | Strongly agree | Do not know |
| --- | --- | --- | --- | --- |
|  |  |  |  |  |

Comments:

2.1 Supply chain infrastructure (e.g. availability of cell material, data storage facilities, etc.) is too unpredictable and fragmented to support scaling-up of the use of NAMs.

| Strongly disagree | Disagree | Agree | Strongly agree | Do not know |
| --- | --- | --- | --- | --- |
|  |  |  |  |  |

Comments:

2.2 Moving NAMs forward from discovery to validation requires specific competencies and quality assurance processes (e.g., certified laboratories) that are too inaccessible to most actors involved in NAM development.

| Strongly disagree | Disagree | Agree | Strongly agree | Do not know |
| --- | --- | --- | --- | --- |
|  |  |  |  |  |

Comments:

2.3 Financial issues in the supply chain (e.g., requirement of expensive testing equipment) are prohibiting the scaling-up of the use of NAMs.

| Strongly disagree | Disagree | Agree | Strongly agree | Do not know |
| --- | --- | --- | --- | --- |
|  |  |  |  |  |

Comments:

2.4 The economics of the supply chain are insufficiently anticipated and managed when popularising NAMs.

| Strongly disagree | Disagree | Agree | Strongly agree | Do not know |
| --- | --- | --- | --- | --- |
|  |  |  |  |  |

Comments:

2.5 The relative costs of animal testing, in comparison to the relative costs of NAMs, disincentivises NAM use.

| Strongly disagree | Disagree | Agree | Strongly agree | Do not know |
| --- | --- | --- | --- | --- |
|  |  |  |  |  |

Comments:

3 **Funding is structured in a way that does not support the popularisation and validation of promising NAMs.**

| Strongly disagree | Disagree | Agree | Strongly agree | Do not know |
| --- | --- | --- | --- | --- |
|  |  |  |  |  |

Comments:

3.1 Discovery is more attractive and comes more naturally to funders than popularisation and validation (e.g., easier to fund, asked for by their constituents, easier to find cost-effective labour to do the work, output is more measurable, higher quantity of projects per unit cost).

| Strongly disagree | Disagree | Agree | Strongly agree | Do not know |
| --- | --- | --- | --- | --- |
|  |  |  |  |  |

Comments:

3.2 Popularisation and validation of NAMs requires continuous and uninterrupted funding, which is hard for funders to provide (e.g. expense, monitoring).

| Strongly disagree | Disagree | Agree | Strongly agree | Do not know |
| --- | --- | --- | --- | --- |
|  |  |  |  |  |

Comments:

3.3 Funding cycles are too short or out of step with current regulatory priorities for popularisation and validation of NAMs that meet regulators’ needs.

| Strongly disagree | Disagree | Agree | Strongly agree | Do not know |
| --- | --- | --- | --- | --- |
|  |  |  |  |  |

Comments:

**Additional comments/Visualisations**

# Workbook – Theme 2

# Creating more promising NAMs

This workbook contains 18 hypotheses derived from the dataset collected in the first phase of CHANGE.

We have developed this workbook to collect your individual feedback on the hypotheses, and to refine our initial theories about how the regulatory toxicology system functions around NAMs.

*Content of the workbook:*

- An overview of the 18 hypotheses (four top-level hypotheses with sub-hypotheses).
- Each hypothesis is presented separately with a 5-point Likert scale for rating your agreement with the hypothesis and space to include comments or visualisations specific to the hypothesis.
- A blank page at the end for other comments/visualisations.

**Overview of the 18 hypotheses**

1. **The actors** who make **the discovery are not necessarily the actors who should do the popularisation and validation.**
   1.1. The specific goals of academics do not align with the specific requirements of popularisation and validation for regulatory purposes.
   1.2. We are lacking mediators between discovery, popularisation and validation of NAMs.
   1.3. Lack of awareness of scientific possibilities by regulators and lack of awareness of regulatory need by academics hampers the creation of a pool of promising NAMs.
   1.4. The publishability and citability of discovery work is much higher than the publishability and citability of popularisation and validation work.
   1.5. There is a disconnect between the reporting checklists and publishing practices employed in academic research and the information needs of regulators (e.g. insufficient information for validity assessment, culture and practices of publishing out of alignment with needs of information users, salami-slicing of data sets).
2. **There are insufficient pull factors for industry to discover, make publicly available and popularise NAMs.**
   2.1. New markets and consumer demands for products will motivate industry to discover, popularise, and validate NAMs (e.g., trends for vegetarian / vegan food or cosmetic products).
   2.2. Anticipated profit (through financial, time, or cost savings) will support the emergence of a well-functioning NAM-based chemical testing industry.
   2.3. Once bitten, twice shy: Industry bases their expectations of regulatory NAM acceptance on previous negative experiences (e.g. dossier rejection).
3. **Inappropriate strategies for managing conflicts of interests inhibit innovation and co-discovery of promising NAMs.**
   3.1. Lack of mechanisms for securing collective agreement about how to act in the context of conflicting values, preferences, and goals around NAM use prevents effective use of NAMs.
   3.2. Removing obstacles and increasing incentives for regulators to work with non-regulatory actors engaged in NAM discovery and popularisation (e.g. academics, industry, NGOs) creates conflicts of interest that reduce the effective use of NAMs.
4. **Political priorities and interests and thus, requirements for risk analysis, are fast-changing over time (different time periods, “trendy” topics) and place (different regulatory frameworks).**
   4.1. Agency shifts in priority are unpredictable and fast-changing, undermining longitudinal (sustained, focused) work on NAMs.
   4.2. Political priorities and interests and scientific innovation cycles are out of sync.
   4.3. Too much focus on specific requirements of an established silo (e.g., regulatory framework / regulation scenario) too early in the discovery and development process inhibits the creation of a pool of promising NAMs.
   4.4. Variation in processes, standards, and terminology in different regulatory contexts increases redundancy in NAM discovery and development.
3. **The actors who make the discovery are not necessarily the actors who should do the popularisation and validation.**

| Strongly disagree | Disagree | Agree | Strongly agree | Do not know |
| --- | --- | --- | --- | --- |
|  |  |  |  |  |

Comments:

1.1 The specific goals of academics do not align with the specific requirements of popularisation and validation for regulatory purposes.

| Strongly disagree | Disagree | Agree | Strongly agree | Do not know |
| --- | --- | --- | --- | --- |
|  |  |  |  |  |

Comments:

1.2 We are lacking mediators between discovery, popularisation and validation of NAMs.

| Strongly disagree | Disagree | Agree | Strongly agree | Do not know |
| --- | --- | --- | --- | --- |
|  |  |  |  |  |

Comments:

1.3 Lack of awareness of scientific possibilities by regulators and lack of awareness of regulatory need by academics hampers the creation of a pool of promising NAMs.

| Strongly disagree | Disagree | Agree | Strongly agree | Do not know |
| --- | --- | --- | --- | --- |
|  |  |  |  |  |

Comments:

1.4 The publishability and citability of discovery work is much higher than the publishability and citability of popularisation and validation work.

| Strongly disagree | Disagree | Agree | Strongly agree | Do not know |
| --- | --- | --- | --- | --- |
|  |  |  |  |  |

Comments:

1.5 There is a disconnect between the reporting checklists and publishing practices employed in academic research and the information needs of regulators (e.g. insufficient information for validity assessment, culture and practices of publishing out of alignment with needs of information users, salami-slicing of data sets).

| Strongly disagree | Disagree | Agree | Strongly agree | Do not know |
| --- | --- | --- | --- | --- |
|  |  |  |  |  |

Comments:

4. **There are insufficient pull factors for industry to discover, make publicly available and popularise NAMs.**

| Strongly disagree | Disagree | Agree | Strongly agree | Do not know |
| --- | --- | --- | --- | --- |
|  |  |  |  |  |

Comments:

2.1 New markets and consumer demands for products will motivate industry to discover, popularise, and validate NAMs (e.g., trend for vegetarian / vegan food or cosmetic products).

| Strongly disagree | Disagree | Agree | Strongly agree | Do not know |
| --- | --- | --- | --- | --- |
|  |  |  |  |  |

Comments:

2.2 Anticipated profit (through financial, time, or cost savings) will support the emergence of a well-functioning NAM-based chemical testing industry.

| Strongly disagree | Disagree | Agree | Strongly agree | Do not know |
| --- | --- | --- | --- | --- |
|  |  |  |  |  |

Comments:

2.3 Once bitten, twice shy: Industry bases their expectations of regulatory NAM acceptance on previous negative experiences (e.g.

dossier rejection).

| Strongly disagree | Disagree | Agree | Strongly agree | Do not know |
| --- | --- | --- | --- | --- |
|  |  |  |  |  |

Comments:

5. **Inappropriate strategies for managing conflicts of interests inhibit innovation and co-discovery of promising NAMs.**

| Strongly disagree | Disagree | Agree | Strongly agree | Do not know |
| --- | --- | --- | --- | --- |
|  |  |  |  |  |

Comments:

3.1 Lack of mechanisms for securing collective agreement about how to act in the context of conflicting values, preferences, and goals around NAM use prevents effective use of NAMs.

| Strongly disagree | Disagree | Agree | Strongly agree | Do not know |
| --- | --- | --- | --- | --- |
|  |  |  |  |  |

Comments:

3.2 Removing obstacles and increasing incentives for regulators to work with non-regulatory actors engaged in NAM discovery and popularisation (e.g. academics, industry, NGOs) creates conflicts of interest that reduce the effective use of NAMs.

| Strongly disagree | Disagree | Agree | Strongly agree | Do not know |
| --- | --- | --- | --- | --- |
|  |  |  |  |  |

Comments:

6. **Political priorities and interests and thus, requirements for risk analysis, are fast-changing over time (different time periods, “trendy” topics) and place (different regulatory frameworks).**

| Strongly disagree | Disagree | Agree | Strongly agree | Do not know |
| --- | --- | --- | --- | --- |
|  |  |  |  |  |

Comments:

4.1 Agency shifts in priority are unpredictable and fast-changing, undermining longitudinal (sustained, focused) work on NAMs.

| Strongly disagree | Disagree | Agree | Strongly agree | Do not know |
| --- | --- | --- | --- | --- |
|  |  |  |  |  |

Comments:

4.2 Political priorities and interests and scientific innovation cycles are out of sync.

| Strongly disagree | Disagree | Agree | Strongly agree | Do not know |
| --- | --- | --- | --- | --- |
|  |  |  |  |  |

Comments:

4.3 Too much focus on specific requirements of an established silo (e.g., regulatory framework / regulation scenario) too early in the discovery and development process inhibits the creation of a pool of promising NAMs.

| Strongly disagree | Disagree | Agree | Strongly agree | Do not know |
| --- | --- | --- | --- | --- |
|  |  |  |  |  |

Comments:

4.4 Variation in processes, standards, and terminology in different regulatory contexts increases redundancy in NAM discovery and development.

| Strongly disagree | Disagree | Agree | Strongly agree | Do not know |
| --- | --- | --- | --- | --- |
|  |  |  |  |  |

Comments:

**Additional comments/Visualisations**

# Workbook - Theme 3

# Understanding and using evidence from NAMs in regulation

The workbook contains 19 hypotheses derived from the dataset collected in the first phase of CHANGE.

We have developed this workbook to collect your individual feedback on these hypotheses, and to refine our initial theories about how the regulatory toxicology system functions around NAMs.

*Content of the workbook:*

- An overview of the 19 hypotheses three top-level hypotheses with sub-hypotheses)
- Each hypothesis presented separately with a 5-point Likert scale for rating your agreement with the hypothesis and space to include comments or visualisations specific to the hypothesis.
- A blank page at the end for other comments/visualisations.

**Overview of the 19 hypotheses**

1. **The necessary familiarity and new sets of knowledge and skills for NAM use are not sufficiently available in the system**
   1.1. Organisational structures prohibit access to, sharing and development of NAM-related skills and knowledge.
   1.2. Insufficient attention is given to the appropriateness of the education to the information needs of the actor (e.g. high-level decision-makers do not have capacity to absorb or even need the technical knowledge of NAM creators).
   1.3. There is insufficient capacity in scientific communication and knowledge transfer (e.g., scientific communication for different audiences, technical writing and reporting) in relation to NAMs.
   1.4. Lack of investment and support by organisations and governments means the pool of capable toxicologists is too small to support transition to NAMs.
   1.5. There is a lack of accounting for the importance of non- scientific knowledge and skills (e.g., legal, commercial, logistical) in relation to NAMs.
   1.6. There is insufficient facility in scientific reasoning (e.g., critical thinking, evaluating the strength of evidence or uncertainty) in relation to NAMs.
2. **Structural issues with NAM evidence make NAMs innately challenging to use in decision-making.**
   2.1. Complex endpoints require batteries of NAMs, which in turn requires ways to overcome statistical false positive rates.
   2.2. The multitude of ways of using NAMs together with the expectations of relying on them in the decision-making process (e.g., challenge of using NAMs as evidence for low risk / safety assessment) are inhibiting the effective use of NAMs.
   2.3. A NAM-based regulatory system is more risk-based, rather than hazard-based.
   2.4. There is a rigidity in standardisation and harmonisation, which facilitates the application of NAMs in decision-making (e.g., due to the improved predictability), but which conflicts with the pace and variability of NAM discovery.
   2.5. NAM evidence is innately uncertain (often indirect and inconsistent, etc.) but decision-making processes cannot currently accommodate uncertain evidence of risk.
3. **Different beliefs and conceptions of what makes a study "valid" across the system stops NAMs being used in regulation**
   3.1. Rapid discovery of NAMs prevents development of familiarity and confidence in them as evidence for application of NAMs in decision making.
   3.2. Due to the rapid and ongoing discovery of NAMs without further development and validation, the focus on retrospective study validation must increase.
   3.3. Different beliefs and conceptions as to what makes a study valid impedes the efficient information flow across different actors in the system.
   3.4. Reliance on expert judgments for validity assessment has substantial limitations (e.g., lack of transparency, over-influenced by “loud voices,” not repeatable or consistent).
   3.5. Consensus approaches for defining standards have substantial limitations (e.g., lack of transparency, over-influenced by "loud voices," not repeatable or consistent).
7. **The necessary familiarity and new sets of knowledge and skills for NAM use are not sufficiently available in the system.**

| Strongly disagree | Disagree | Agree | Strongly agree | Do not know |
| --- | --- | --- | --- | --- |
|  |  |  |  |  |

Comments:

a. Organisational structures prohibit access to, sharing and development of NAM-related skills and knowledge.

| Strongly disagree | Disagree | Agree | Strongly agree | Do not know |
| --- | --- | --- | --- | --- |
|  |  |  |  |  |

Comments:

b. Insufficient attention is given to the appropriateness of the education to the information needs of the actor (e.g. high-level decision-makers do not have capacity to absorb or even need the technical knowledge of NAM creators).

| Strongly disagree | Disagree | Agree | Strongly agree | Do not know |
| --- | --- | --- | --- | --- |
|  |  |  |  |  |

Comments:

c. There is insufficient capacity in scientific communication and knowledge transfer (e.g., scientific communication for different audiences, technical writing and reporting) in relation to NAMs.

| Strongly disagree | Disagree | Agree | Strongly agree | Do not know |
| --- | --- | --- | --- | --- |
|  |  |  |  |  |

Comments:

d. Lack of investment and support by organisations and governments means the pool of capable toxicologists is too small to support transition to NAMs.

| Strongly disagree | Disagree | Agree | Strongly agree | Do not know |
| --- | --- | --- | --- | --- |
|  |  |  |  |  |

Comments:

e. There is a lack of accounting for the importance of non- scientific knowledge and skills (e.g., legal, commercial, logistical) in relation to NAMs.

| Strongly disagree | Disagree | Agree | Strongly agree | Do not know |
| --- | --- | --- | --- | --- |
|  |  |  |  |  |

Comments:

f. There is insufficient facility in scientific reasoning (e.g., critical thinking, evaluating the strength of evidence or uncertainty) in relation to NAMs.

| Strongly disagree | Disagree | Agree | Strongly agree | Do not know |
| --- | --- | --- | --- | --- |
|  |  |  |  |  |

Comments:

8. **Structural issues with NAM evidence make NAMs innately challenging to use in decision-making.**

| Strongly disagree | Disagree | Agree | Strongly agree | Do not know |
| --- | --- | --- | --- | --- |
|  |  |  |  |  |

Comments:

2.1 Complex endpoints require batteries of NAMs, which in turn requires ways to overcome statistical false positive rates.

| Strongly disagree | Disagree | Agree | Strongly agree | Do not know |
| --- | --- | --- | --- | --- |
|  |  |  |  |  |

Comments:

2.2 The multitude of ways of using NAMs together with the expectations of relying on them in the decision-making process (e.g., challenge of using NAMs as evidence for low risk / safety assessment) are inhibiting the effective use of NAMs.

| Strongly disagree | Disagree | Agree | Strongly agree | Do not know |
| --- | --- | --- | --- | --- |
|  |  |  |  |  |

Comments:

2.3 A NAM-based regulatory system is more risk-based, rather than hazard-based.

| Strongly disagree | Disagree | Agree | Strongly agree | Do not know |
| --- | --- | --- | --- | --- |
|  |  |  |  |  |

Comments:

2.4 There is a rigidity in standardisation and harmonisation, which facilitates the application of NAMs in decision-making (e.g., due to the improved predictability), but which conflicts with the pace and variability of NAM discovery.

| Strongly disagree | Disagree | Agree | Strongly agree | Do not know |
| --- | --- | --- | --- | --- |
|  |  |  |  |  |

Comments:

2.5 NAM evidence is innately uncertain (often indirect and inconsistent, etc.) but decision-making processes cannot currently accommodate uncertain evidence of risk.

| Strongly disagree | Disagree | Agree | Strongly agree | Do not know |
| --- | --- | --- | --- | --- |
|  |  |  |  |  |

Comments:

3 **Different beliefs and conceptions of what makes a study "valid" across the system stops NAMs being used in regulation.**

| Strongly disagree | Disagree | Agree | Strongly agree | Do not know |
| --- | --- | --- | --- | --- |
|  |  |  |  |  |

Comments:

3.1 Rapid discovery of NAMs prevents development of familiarity and confidence in them as evidence for application of NAMs in decision making.

| Strongly disagree | Disagree | Agree | Strongly agree | Do not know |
| --- | --- | --- | --- | --- |
|  |  |  |  |  |

Comments:

3.2 Due to the rapid and ongoing discovery of NAMs without further development and validation, the focus on retrospective study validation must increase.

| Strongly disagree | Disagree | Agree | Strongly agree | Do not know |
| --- | --- | --- | --- | --- |
|  |  |  |  |  |

Comments:

3.3 Different beliefs and conceptions as to what makes a study valid impedes the efficient information flow across different actors in the system.

| Strongly disagree | Disagree | Agree | Strongly agree | Do not know |
| --- | --- | --- | --- | --- |
|  |  |  |  |  |

Comments:

3.4 Reliance on expert judgments for validity assessment has substantial limitations (e.g., lack of transparency, over-influenced by “loud voices,” not repeatable or consistent).

| Strongly disagree | Disagree | Agree | Strongly agree | Do not know |
| --- | --- | --- | --- | --- |
|  |  |  |  |  |

Comments:

3.5 Consensus approaches for defining standards have substantial limitations (e.g., lack of transparency, over-influenced by "loud voices," not repeatable or consistent).

| Strongly disagree | Disagree | Agree | Strongly agree | Do not know |
| --- | --- | --- | --- | --- |
|  |  |  |  |  |

Comments:

**Additional comments/Visualisations**
